# Infection with SARS-CoV-2 can cause pancreatic impairment

**DOI:** 10.1038/s41392-024-01796-2

**Published:** 2024-04-12

**Authors:** Wei Deng, Linlin Bao, Zhiqi Song, Ling Zhang, Pin Yu, Yanfeng Xu, Jue Wang, Wenjie Zhao, Xiuqin Zhang, Yunlin Han, Yanhong Li, Jiangning Liu, Qi Lv, Xujian Liang, Fengdi Li, Feifei Qi, Ran Deng, Siyuan Wang, Yibai Xiong, Ruiping Xiao, Hongyang Wang, Chuan Qin

**Affiliations:** 1 NHC Key Laboratory of Comparative Medicine, Beijing Key Laboratory for Animal Models of Emerging and Remerging Infectious Diseases, Institute of Laboratory Animal Science, Chinese Academy of Medical Sciences and Comparative Medicine Center, Peking Union Medical College, Beijing, 100021 China; 2 Institute of Molecular Medicine, College of Future Technology, Peking University, Beijing, 100871 China; 3 Beijing Key Laboratory of Cardiometabolic Molecular Medicine, Peking University, Beijing, 100871 China; 4 State Key Laboratory of Biomembrane and Membrane Biotechnology, Peking-Tsinghua Center for Life Sciences, Beijing, 100871 China; 5 Chinese Academy of Engineering, Eastern Hepatobiliary Surgery Hospital, 225 Changhai Road, Yangpu District Shanghai, 200438 China; 6 International Co-operation Laboratory on Signal Transduction, Eastern Hepatobiliary Surgery Institute, Second Military Medical University, Shanghai, 200438 PR China; 7 National Laboratory for Oncogenes and Related Genes, Cancer Institute of Shanghai Jiao Tong University, Shanghai, 200441 PR China; 8 Changping National laboratory (CPNL), Beijing, 102206 China; 9 State Key Laboratory of Respiratory Health and Multimorbidity, National Health Commission of the People’s Republic of China, Beijing, PR China

**Keywords:** Infectious diseases, Endocrine system and metabolic diseases, Health care

## Abstract

Evidence suggests associations between COVID-19 patients or vaccines and glycometabolic dysfunction and an even higher risk of the occurrence of diabetes. Herein, we retrospectively analyzed pancreatic lesions in autopsy tissues from 67 SARS-CoV-2 infected non-human primates (NHPs) models and 121 vaccinated and infected NHPs from 2020 to 2023 and COVID-19 patients. Multi-label immunofluorescence revealed direct infection of both exocrine and endocrine pancreatic cells by the virus in NHPs and humans. Minor and limited phenotypic and histopathological changes were observed in adult models. Systemic proteomics and metabolomics results indicated metabolic disorders, mainly enriched in insulin resistance pathways, in infected adult NHPs, along with elevated fasting C-peptide and C-peptide/glucose ratio levels. Furthermore, in elder COVID-19 NHPs, SARS-CoV-2 infection causes loss of beta (β) cells and lower expressed-insulin in situ characterized by islet amyloidosis and necrosis, activation of α-SMA and aggravated fibrosis consisting of lower collagen in serum, an increase of pancreatic inflammation and stress markers, ICAM-1 and G3BP1, along with more severe glycometabolic dysfunction. In contrast, vaccination maintained glucose homeostasis by activating insulin receptor α and insulin receptor β. Overall, the cumulative risk of diabetes post-COVID-19 is closely tied to age, suggesting more attention should be paid to blood sugar management in elderly COVID-19 patients.

## Introduction

COVID-19, in addition to causing respiratory failure, has led to multi-organ complications, including those involving the pancreas and endocrine system.^1–3^ Diabetes is a significant risk factor for COVID-19 susceptibility.^4,5^ According to a recent report based on 72,314 COVID-19 cases in China, people with preexisting diabetes had the second-highest case-fatality rate.^6^ Larger studies have indicated that diabetic patients with COVID-19 are at a higher risk of experiencing severe complications requiring intensive care unit (ICU) admission.^7^ Additionally, experimental data suggest SARS-CoV-2 infiltration of exocrine and endocrine pancreatic cells both in vitro and in vivo.^8–11^ However, it remains unclear whether the pancreatic lesion is permanent or varies with age. More extensive research is needed to confirm the relationship between diabetes and COVID-19, as well as to determine if direct β cell infection or impairment via indirect mechanisms occurs in patients of different ages.

Vaccine is another possible factor that may cause pancreatic damage due to COVID-19. Several highly effective SARS-CoV-2 vaccines have been developed for COVID-19 prevention; however, the potential side effects of these newly developed vaccines still require comprehensive understanding. Further research is currently being conducted on the protective and adverse effects of vaccines on human health. Laboratory data is crucial in identifying both short-term and long-term vaccine side effects. Although rare acute side effects have also been reported, such as acute pancreatitis after vaccine injection;^12–15^ It still remains uncertain whether vaccine injection results in permanent or temporary changes in pancreas and glucose metabolism.

Our research team works at the Institute of Laboratory Animal Science, Chinese Academy of Medical Sciences, and Comparative Medicine Center, where our pathology laboratory stores valuable animal experimental specimens and wax blocks from different experiments throughout the year for long-term research and study. Since the outbreak of COVID-19 at the end of 2019, our team has constructed a series of animal models of SARS-CoV-2 infection to study the pathogenic mechanisms and transmission route of SARS-CoV-2 to evaluate the safety and efficacy of vaccines and therapeutic drugs under testing. In this study, we retrospectively collected pancreatic tissue samples from 188 rhesus macaques, including 67 SARS-CoV-2 infected non-human primates (NHPs) models and 121 vaccinated and infected NHPs from different trials from 2020 to 2023. Among these animals, the prototypic SARS-CoV-2 strain-related animals, including 9 prototypic SARS-CoV-2 strain-infected adult NHPs model, 6 prototypic SARS-CoV-2 strain-infected elder NHPs model, and 35 COVID-19 vaccines immunized and prototypic SARS-CoV-2 strain-infected adult NHPs models were further selected as the groups for in-depth study. Above COVID-19 vaccines provide well immune protection effect on animals after vaccination, and are recommended for further clinical research and marketing. Specimens and wax blocks of pancreatic tissue from three uninfected adult NHPs and three elder NHPs stored in our lab were collected for the adult control group and the elder control group. Compared among these groups, we want to evaluate SARS-CoV-2 infection-related pancreatic impairment and disturbance of glucose metabolism and explore whether COVID-19 vaccines could affect the homeostasis of glucose metabolism.

To better comprehend the potential effects of SARS-CoV-2 on the pancreas of COVID-19 patients of varying ages, we conducted a detailed investigation using those autopsied samples from SARS-CoV-2-infected NHPs. By employing multi-label immunofluorescence analysis with various biomarkers, we were able to discern several key features: the distribution of SARS-CoV-2 receptors within the pancreas, the extent of SARS-CoV-2 infiltration in both the exocrine and endocrine regions of the pancreas and the degree of damage or dysfunction in pancreatic islets caused by SARS-CoV-2 in elder NHPs. Lastly, through the integration of systemic proteomics, lipidomics, and metabolomics data obtained from NHP sera, along with direct visualization of protein expression, we identified distinct metabolic profiles among SARS-CoV-2-infected adults, elders, and vaccinated NHPs.

## Results

### SARS-CoV-2 can directly infect NHP and human pancreatic microvasculature and most types of exocrine and endocrine cells and in situ tissue

Several reports have demonstrated that SARS-CoV-2 is localized to pancreatic cells, such as pancreatic ductal epithelium, endothelial cells, acinar cells, mesenchymal cells, and β cells.^8–11^ Currently, there is a lack of experimental data on the infiltration and distribution of SARS-CoV-2 in exocrine and endocrine pancreatic cells on the same slice in situ. In this study, we aimed to investigate the relationship between SARS-CoV-2 and different types of islet cells, including alpha cells (α cells), β cells, delta cells (δ cells), and pancreatic polypeptide cells (PP cells), which play key roles in regulating endocrine function and glucose metabolism of pancreatic islets in NHPs and COVID-19 subjects. Human pancreatic tissue samples were used as a comparison to (1) compare the histological similarities and differences between human and NHPs pancreatic tissues and (2) compare the similarities and differences in histopathological states after SARS-CoV-2 infection and further verify the results found on NHPs pancreatic tissues.

Angiotensin-converting enzyme 2 (ACE2) and a plasma membrane-associated type II transmembrane serine protease (TMPRSS2) are two of the most important mediators for SARS-CoV-2 entry.^16,17^ SARS-CoV-2 entry into cells via the ACE2 receptor requires S protein priming by TMPRSS2.^17^ Neuropilin 1 (NRP1) is also an important host co-factor for SARS-CoV-2 infection, and recent research demonstrated that the highly expressed neuropilin 1 receptor is critical for viral entry.^18,19^ The expression of these viral receptors in the pancreas is uncertain, as contradictory results have been reported. Firstly, the protein expression and distribution of the main SARS-CoV-2 entry factors, ACE2, TMPRSS2, and NRP1, in combination with insulin, a β cell marker, glucagon, and α cell maker, were tested and observed by multi-label immunofluorescence in both the autopsy and NHP samples (Supplementary Fig. 1a–c). Different from previous methodologies, by multi-label immunofluorescence, we demonstrated that ACE2, NRP1, and TMPRSS2 proteins were generally expressed within β and α cells in both samples, consistent with their mRNA expression.^11^ Furthermore, through quantitative analysis (Supplementary Fig. 1d–h), we found significantly increased expression of NRP1 and TMPRSS1 protein after SARS-CoV-2 infection in elder NHPs compared with elder controls (Supplementary Fig. 1e). And a significantly higher co-expression proportion of NRP1 or TMPRSS1 and insulin was observed (Supplementary Fig. 1g, h), while that of ACE2 and insulin was decreased in SARS-CoV-2-infected elder NHPs (Supplementary Fig. 1f).^20,21^ Taken together, in response to SARS-CoV-2 invasion, β cells may inhibit ACE2 expression as a negative feedback regulation, TMPRSS1 expression is increased, and NRP1 expression, as an alternative receptor, is stimulated.

**Fig. 1:**
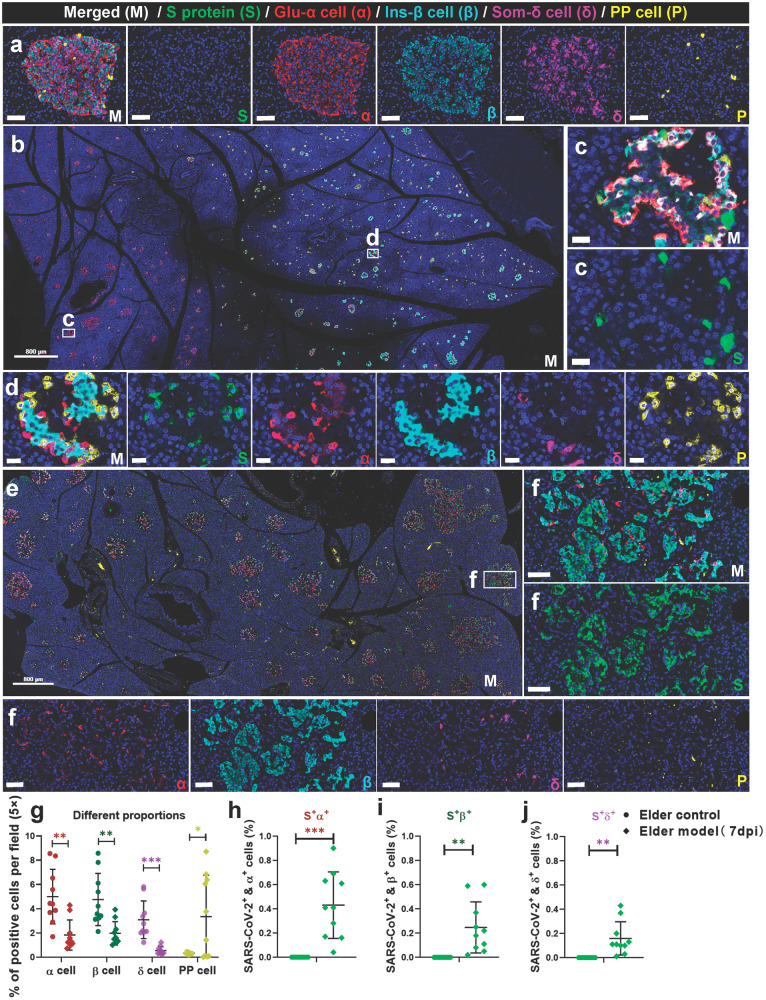
SARS-CoV-2 directly infects the pancreatic islets of non-human primates (NHPs). **a** Representative multi-label IF image from the elder control NHP sample was stained for SARS-CoV-2 S1 protein (S, green), glucagon (α, red), insulin (β, cyan), somatostatin (δ, magenta), and polypeptide (P, yellow). Scale bars, 50 μm. **b**–**d** Pancreatic tissue section from one SARS-CoV-2-infected elder NHP sample was stained by the same panel of multi-label IF, showing the co-localization of S and P. Scale bars, 800 μm. **c**, **d** Representative multi-label IF image from the magnified section of (**b**). Inset highlights SARS-CoV-2 viral antigen co-localized with islet endocrine cells. Scale bars, 20 μm. **e** Pancreatic tissue section from another SARS-CoV-2-infected elder NHP sample was stained by the same panel of multi-label IF, showing the co-localization of S and markers of various islet endocrine cells. Scale bars, 800 μm. **f** Representative multi-label IF image from the magnified section of (**e**). Inset highlights co-expression of SARS-CoV-2 S protein (S, green) with glucagon (α, red), insulin (β, cyan), somatostatin (δ, magenta), and polypeptide (P, yellow). Scale bars, 100 μm. **g**–**j** Quantification of the percentage of glucagon^+^ α, insulin^+^ β, somatostatin^+^ δ, and polypeptide^+^ PP cells, as well as SARS-CoV-2 S protein^+^glucagon^+^ α, S protein^+^insulin^+^ β, and S protein^+^somatostatin^+^ δ cells, in the elder control NHPs (3 slides) and elder COVID-19 model NHPs(4 slides) (*n* = 10 images examined from all slides/group). Data are presented as mean ± SD. *p* Values were calculated by paired or unpaired two-tailed Student’s *t* test. *p < 0.05, **p < 0.01, and ***p < 0.001

**Fig. 2:**
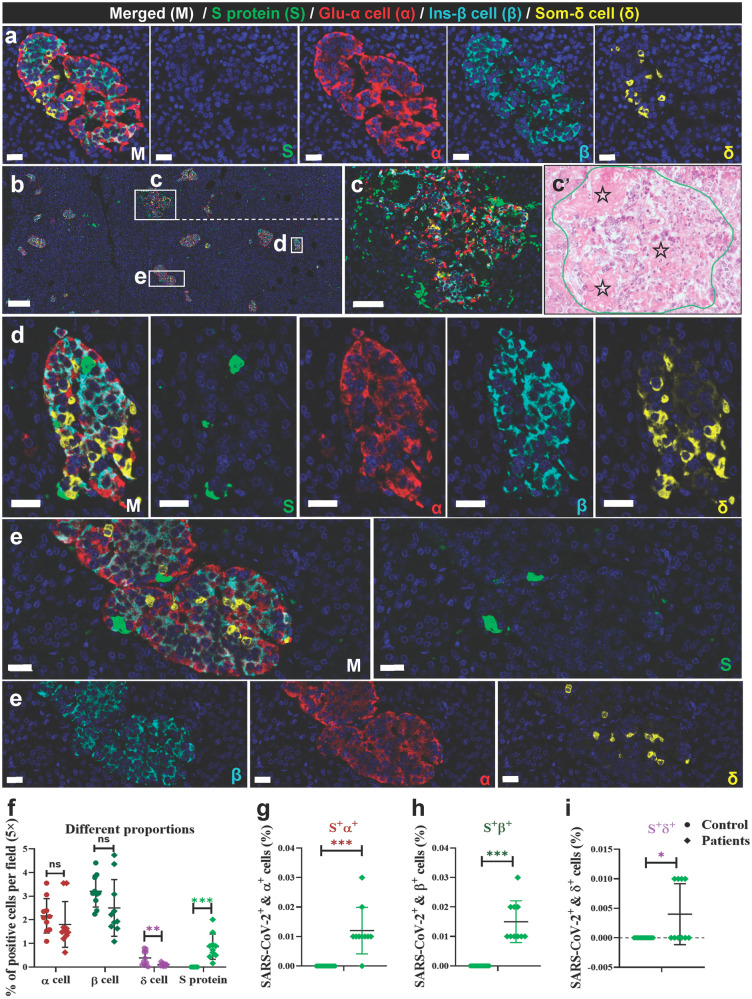
SARS-CoV-2 directly infects the human pancreatic islet cells. **a** Pancreatic tissue sections from the deceased control subject were stained by multi-label IF for SARS-CoV-2 S1 protein (S, green), glucagon (α, red), insulin (β, cyan), and somatostatin (δ, yellow) (*n* = 10 images examined in total). Scale bars, 50 μm. **b** Pancreatic tissue sections from the COVID-19 autopsy samples (the prototypic SARS-CoV-2 strain-infected) were stained by multi-label IF for SARS-CoV-2 S1 protein (S, green), glucagon (α, red), insulin (β, cyan), and somatostatin (δ, yellow) (*n* = 10 images examined in total). Scale bars, 200 μm. **c** Representative multi-label IF image from the magnified section of (**b**). Inset highlights SARS-CoV-2 viral antigen scattered in a severely damaged and necrotic islet. Scale bars, 50 μm. **c** The serial section from the same pancreatic tissue stained for H&E and the same necrotic islet was magnified and circled (blue) to clearly observe its pathological changes. The islet structure is disorganized, with cellular swelling and degeneration observed in the internal regions. Cytoplasmic eosinophilia is intensified, and nuclear swelling is evident. The pentagon-marked area (☆) indicates significant necrosis of islet cells, with nuclear pyknosis, karyolysis, and loss of original cellular structure, leaving only homogeneously stained eosinophilic areas. **d**, **e** Representative multi-label IF image from the magnified section of (a). Inset highlights SARS-CoV-2 viral antigen co-localized with islet endocrine cells. Scale bars, 50 μm. COVID-19, coronavirus disease 2019; H&E, hematoxylin and eosin; SARS-CoV-2, severe acute respiratory syndrome coronavirus 2. **f**–**i** Quantification of the percentage of glucagon^+^ α, insulin^+^ β, somatostatin^+^ δ, and SARS-CoV-2 S protein^+^ cells, as well as S protein^+^glucagon^+^ α, S protein^+^insulin^+^ β, and S protein^+^somatostatin^+^ δ cells, in control human pancreatic tissues and the COVID-19 patients pancreatic tissues (*n* = 10 images/group). Data are presented as mean ± SD. *p* Values were calculated by paired or unpaired two-tailed Student’s *t* test. *p < 0.05, **p < 0.01, and ***p < 0.001

The SARS-CoV-2 receptors allow for viral infiltration. The specificity of the commercial SARS-CoV-2 antibody has been tested and validated in our recent studies.^22–24^ Firstly, we examined SARS-CoV-2 S protein immunopositivity in all pancreatic sections from autopsy and NHP samples. Further, the presence of SARS-CoV-2 RNA was verified by in situ hybridization. SARS-CoV-2 initially invaded the endothelial cells of the microvasculature in the mildly damaged pancreas and then dispersed among the exocrine and endocrine pancreatic cells. Importantly, S protein immunopositivity samples had a higher frequency and expression in SARS-CoV-2-infected elder NHPs than adult NHPs. Next, we tested the characteristics of SARS-CoV-2 distribution in the islets. Compared with the elder control group (Fig. 1a), glucagon^+^ α cells (reduced by 63.49%), insulin^+^ β cells (reduced by 58.66%), and somatostatin^+^ δ cells (reduced by 82.33%) were significantly decreased, while pancreatic polypeptide^+^ PP cells were significantly increased in SARS-CoV-2-infected elder NHPs (Fig. 1b–f). Moreover, S^+^insulin^+^ β, S^+^glucagon^+^ α, S^+^somatostatin^+^ δ, and S^+^polypeptide^+^ PP cells demonstrated that SARS-CoV-2 directly infected various types of endocrine cells (Fig. 1g–j). Furthermore, the same panel of multi-label immunofluorescence was examined in pancreatic sections of humans. Compared with the control pancreatic tissues (Fig. 2a), co-staining with pancreatic endocrine and non-endocrine markers confirmed the presence of SARS-CoV-2 antigen (S protein) and its relationship with glucagon-secreting α cells, insulin-secreting β cells, and somatostatin-secreting δ cells in the pancreas of autopsy samples (Fig. 2b–e). Quantitative analysis demonstrated that, compared with the control pancreatic tissues, somatostatin^+^ δ cells (reduced by 72.47%) were significantly decreased in COVID-19 patients’ samples (Fig. 2f). Consistently, S^+^insulin^+^ β, S^+^glucagon^+^ α and S^+^somatostatin^+^ δ cells demonstrated that SARS-CoV-2 directly infected various types of endocrine cells (Fig. 2g–i). Importantly, HE-stained serial sections of the same sample revealed that several pancreatic islets were undergoing degeneration and necrosis characterized by nuclear pyknosis, karyolysis, and loss of original cellular structure, leaving only homogeneously stained eosinophilic areas (Fig. 2b’). Consistent with these pathological changes, various types of islet cells were significantly impaired and reduced in number.

### SARS-CoV-2 infection induces circumscribed pancreatic phenotypic alterations in adult NHPs and aggravates diabetes-like pathological phenotype in elder NHPs

Firstly, we observed the HE-stained sections of all samples to examine the number and extent of major endocrine and exocrine pancreatic lesions between groups. Compared with the adult control monkeys, pathology from adult COVID-19 models was mild, displaying marginally increased degeneration and inflammatory infiltration.

**Fig. 3:**
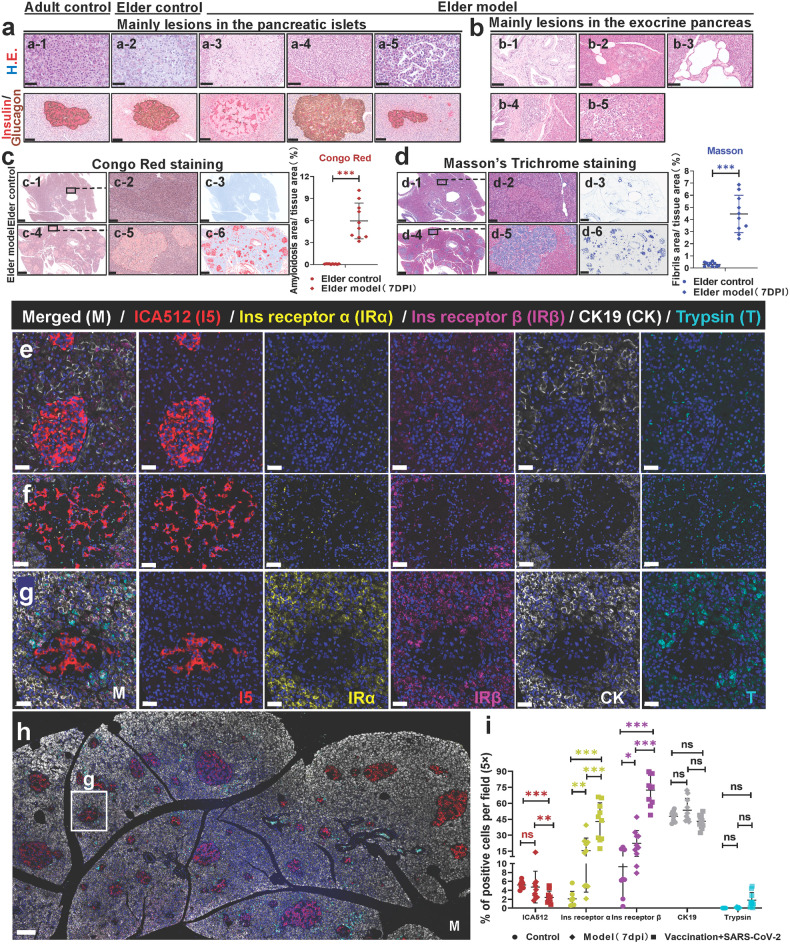
Characteristics of pathological changes in adult and elder COVID-19 NHPs and vaccination triggers insulin receptor activation in the COVID-19 NHP model. **a** The main lesions in the pancreatic islets observed in the elder prototypic SARS-CoV-2 strain-infected model (a-3 to a-5) compared with the adult (a-1) and elder control (a-2) animals. The first line shows the representative images of pancreatic tissue sections stained by H&E. The second line shows the representative IHC images of pancreatic tissue sections stained for insulin (red) and glucagon (brown). (a-1) Normal pancreatic islets and surrounding tissues in the adult control group; (a-2) a few islet cells are swelling of individual pancreatic islets in the elder control group. In the elder prototypic SARS-CoV-2 strain-infected model, (a-3) the number of islets cells is significantly decreased, and a large amount of amyloid substance is deposited in some islets; (a-4) glucagon^+^ alpha cells are increased in individual islets; (a-5) a few islets are atrophy. Scale bars, 50 μm or 100 μm shown in the corresponding images below. **b** The main lesions in the exocrine pancreas observed in the elder prototypic SARS-CoV-2 strain-infected model (b-1 to b-5). (b-1) Exfoliated cells can be observed in a few pancreatic duct lumen; (b-2) hyperplasia of local pancreatic ductal; (b-3) dilation of pancreatic duct; (b-4) pancreatic periductal inflammatory cells infiltrated around the pancreatic duct; (b-5) inflammatory cells infiltrated in the interstitium. The representative images of pancreatic tissue sections stained by H&E. Scale bars, 50 μm or 100 μm shown in the corresponding images below. **c** Qualitative and quantitative analysis by Congo red staining to examine prototypic SARS-CoV-2 strain-infection-elicited islet amyloidosis in the elder COVID-19 model (4 slides) compared with the elder control (3 slides) (*n* = 10 images examined from all slides/group). **d** The serial section was stained by Masson’s trichrome staining, qualitatively and quantitatively, to observe SARS-CoV-2-infection-elicited islet amyloidosis in the elder COVID-19 model infected with the prototypic SARS-CoV-2 strain compared with the elder control (*n* = 10 images examined in total/group). **e**–**h** Representative multi-label IF image from adult control (**a**), prototypic SARS-CoV-2-strain-infected COVID-19 NHP model (**b**), and vaccinated COVID-19 NHP model (**c**, **d**) pancreatic tissue section samples were stained for ICA512 (I5, red), insulin receptor α (IRα, yellow), insulin receptor β (IRβ, magenta), CK19 (CK, white), and trypsin (T, cyan). Scale bars, 20 μm in (**a**–**c**). **c** Representative multi-label IF images from section (**d**). Scale bars, 100 μm in (**d**). **i** Quantification of the percentage of ICA512^+^, insulin receptor α (Ins R α)^+^, insulin receptor β (Ins R β)^+^, CK19^+^, and trypsin^+^ cells in the control NHPs (3 slides), COVID-19 NHP model (3 slides) and vaccinated COVID-19 NHP model (3 slides) (*n* = 10 images examined from all slides /group). Data are presented as mean ± SD. *p* Values were calculated by paired or unpaired two-tailed Student’s *t* test. *p < 0.05, **p < 0.01, and ***p < 0.001

**Fig. 4:**
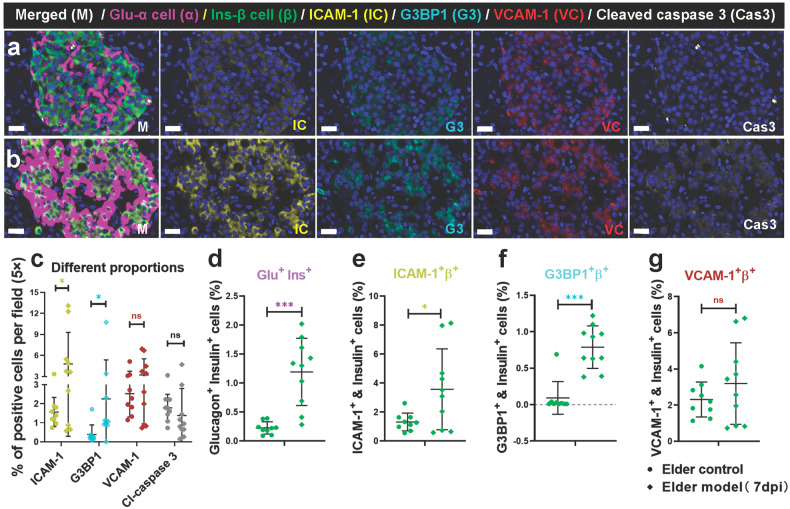
Characteristics of the markers of microvascular damage and cellular stress in elder control and elder COVID-19 model NHPs. **a**, **b** The markers of microvascular damage and cellular stress, ICAM-1 and G3BP1, were elevated in the islets of elder COVID-19 model (**a**) compared with the elder control NHPs (**b**). Representative multi-labels IF image in the pancreas from the elder control samples were stained for glucagon (α, magenta), insulin (β, green), ICAM-1 (IC, yellow), G3BP1 (G3, cyan), VCAM-1 (VC, red) and Cleaved caspase 3 (Cas3, white). Scale bars, 20 μm. **c**–**g** Quantification of the percentage of ICAM-1^+^ cell, G3BP1^+^ cell, VCAM-1^+^ cell, and cleaved caspase 3^+^ cell, as well as glucagon^+^insulin^+^ cell, ICAM-1^+^insulin^+^ cell, G3BP1^+^insulin^+^ cell and VCAM-1^+^insulin^+^ cell in the elder control NHPs (3 slides) and elder COVID-19 model NHPs (4 slides), (*n*=10 images examined from all sildes/group). Data are presented as mean ± SD. *p* values were calculated by unpaired two-tailed Student’s *t* test. *p < 0.05 and ***p < 0.001

**Fig. 5:**
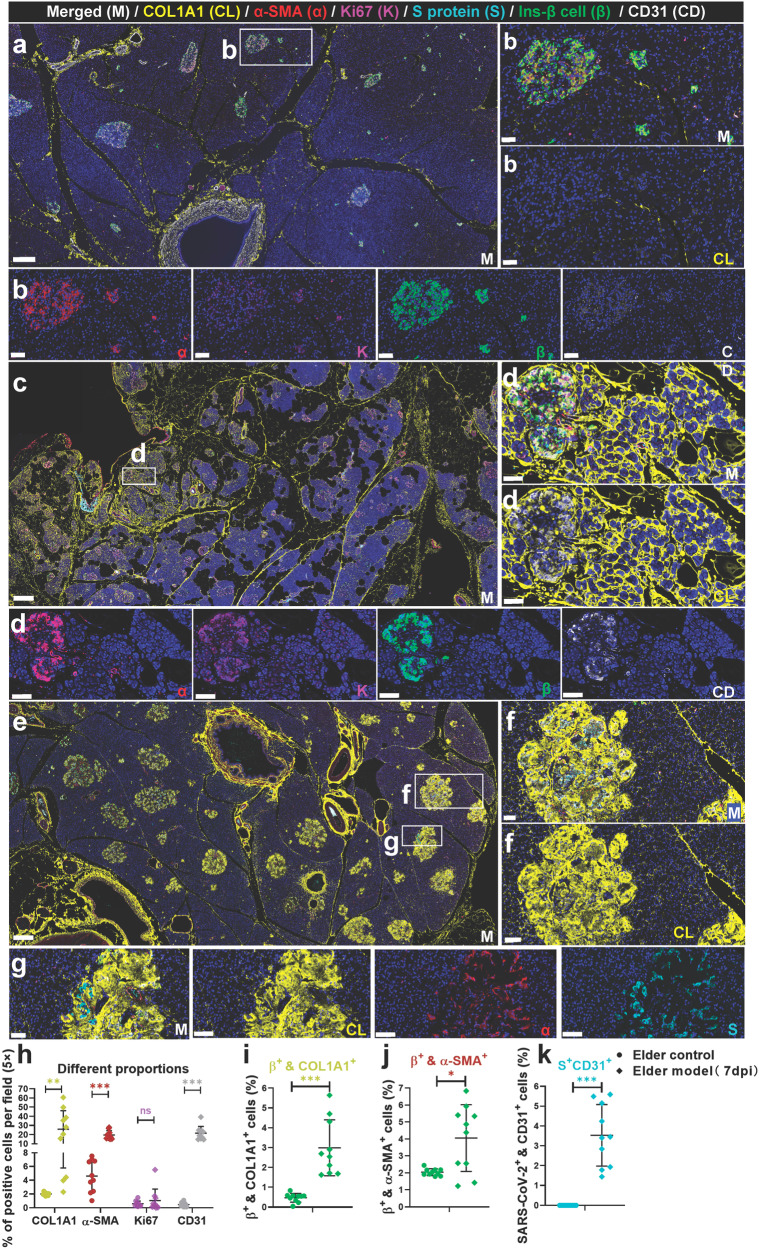
SARS-CoV-2 infection aggregated activation of α-SMA and accumulation of collagen fibers from the NHP pancreatic tissues of the elder COVID-19 model. Pancreatic tissue sections from the elder control NHP samples were stained by multi-label immunofluorescence (IF) for COL1A1 (CL, yellow), α-SMA (α, red), Ki67 (K, magenta), SARS-CoV-2 S1 protein (S, cyan), insulin (β, green), and CD31 (CD, white). Scale bars, 200 μm. Representative multi-label IF image from the magnified section of (**a**). Scale bars, 20 μm. The pancreatic tissue section from one elder NHP model sample was stained by the same multi-label makers in (**a**). Scale bars, 400 μm. Representative multi-label IF image from the magnified section of (**c**). Inset highlights proliferating collagen fibers dividing the exocrine and endocrine pancreatic tissues into various islands. Scale bars, 20 μm. **e** Pancreatic tissue section from another elder prototypic SARS-CoV-2 strain-infected NHP model sample was stained by the same multi-label makers in (**a**). Scale bars, 800 μm. **f**, **g** Representative multi-label IF image from the magnified section of (**e**). Inset highlights co-localization of SARS-CoV-2 viral antigen and accumulated collagen fibers in damaged islet insulin^+^ β cells. Scale bars in **f** 40 μm. Scale bars in **g** 20 μm. **h**–**k** Quantification of the percentage of COL1A1^+^, α-SMA^+^, Ki67^+^, and CD31^+^ cells, as well as insulin^+^COL1A1^+^, insulin^+^ α-SMA^+^, and S protein^+^ CD31^+^ cells, in the elder control NHPs (3 slides) and elder COVID-19 NHP model (4 slides) (*n* = 10 images examined from all slides /group). Data are presented as mean ± SD. *p* Values were calculated by unpaired two-tailed Student’s *t* test. *p < 0.05, **p < 0.01, and ***p < 0.001

Compared with the elder control monkeys, pathological phenotypes from SARS-CoV-2-infected elder monkeys (the prototypic SARS-CoV-2 strain) were more characterized by aggravated degeneration, amyloidosis, necrosis or atrophy in the islets, and increased hyperplasia, dilatation, adipocytes, and inflammatory cell infiltration in the exocrine pancreas (Table 1). By double immunohistochemical staining of insulin (AP-red) and glucagon (DAB-brown), the lesions in the islets of the elder COVID-19 models were clearly shown (Fig. 3a). Compared with the islets in the adult controls (Fig. 3a-1), several islets in the elder controls underwent degeneration, and slight amyloidosis was detected in the islet cells and nuclei (Fig. 3a-2). The islet amyloidosis appeared as homogeneous to faintly fibrillar, pale eosinophilic deposits in the pancreatic islet interstitium. Meantime, insulin^+^ β and glucagon^+^ α cells significantly decreased and were pushed aside (Fig. 3a-3). Islet cell hyperplasia was observed in one case, which was confirmed as glucagon^+^ α cells increased (Fig. 3a-4). Figure 3a-5 shows islet atrophy resulting in a decreased islet volume. Additionally, shedding of cells in the endocrine ducts (Fig. 3b-1), exocrine duct hyperplasia and dilatation (Fig. 3b-2, b-3), and exocrine pancreas degeneration and inflammation (Fig. 3b-4, b-5) were observed in elder COVID-19 models. As islet amyloidosis can be associated with insulin-resistant (Type 2) diabetes mellitus,^25,26^ we preliminarily examined these serial sections by special staining. These amyloids were positively stained using Congo red stain, and the average areas of Congo-red-positive deposition expanded by 5.86% (Fig. 3c). Surprisingly, mostly amyloids were also positively stained using Masson stain, and the average areas expanded by 4.16% (Fig. 3d).

**Table 1:** Summary of pathological changes and SARS-CoV-2 infection percentage in adult and elder COVID-19 NHPs and vaccinated-infected NHPs

Groups (No.) Lesions (degree)	Control (6)		*Rhesus macaque* models of COVID-19 (15)		Inactivated vaccines/subunit (recombinant protein) vaccines + prototypic strain infection (35)
	Adult (3)	Elder (3)	Adult-prototypic strain infection (9)	Elder-prototypic strain infection (6)	
Degeneration of islets	0/3	1/3	1/9	4/6	1/35
Necrosis of islets	0/3	0/3	0/9	3/6	0/35
Telangiectasis in the islets	0/3	1/3	1/9	1/6	3/35
Atrophy of islets	0/3	1/3	0/9	5/6	0/35
Hyperplasia of islet cell	0/3	0/3	0/9	1/6	0/35
Islet amyloidosis	0/3	1/3	0/9	4/6	0/35
Degeneration of exocrine pancreas	1/3	0/3	0/9	3/6	0/35
Shedding cells in the pancreatic exocrine duct	0/3	0/3	2/9	1/6	4/35
Dilatation of pancreatic exocrine duct	0/3	1/3	0/9	1/6	0/35
Inflammatory cell infiltration	0/3	1/3	4/9	4/6	8/35

Notes: The table summarizes the proportion of different lesions, percentage of inflammation in different groups

Previous research has demonstrated that islet amyloidosis is characterized by amyloid fibrils, and disease severity may depend more on the biochemical nature of the amyloid fibrils. In fact, the precursor peptides or intermediate oligomers, rather than the mature amyloid fibrils, are thought to be injurious and cytotoxic agents, at least in islet amyloidosis. The amyloid deposited in the pancreatic islets of human beings and NHPs is derived from islet amyloid peptide and is secreted by β cells. It can be associated with insulin-resistant (type 2) diabetes mellitus. Taken together, SARS-CoV-2 infection induces mild and limited pancreatic phenotypic alterations in adult NHPs and largely aggravates pathological phenotypes perhaps related to diabetes in elder NHPs.

To examine the clinical biochemical indicators associated with pancreatic metabolism, we collected 31 serum samples after overnight fasting from different groups (3 samples from adult control, 3 samples from elder control, 9 samples from the adult model, 9 samples from the elder model, 7 samples from vaccine + adult model) (Supplementary Fig. 2a–f). We observed that NHPs in the adult model exhibited significantly elevated levels of fasting C-peptide and C-peptide/glucose ratio, which are usually used to assess insulin resistance,^27^ compared with that of adult controls. For the elder model, both the expression of C-peptide and glucose had a tendency to increase compared with the elder control while without significant difference (Supplementary Fig. 2a), which may be due to the small sample size in the elder control animals. In general, there was no significant change in the fasting glucose and fasting insulin levels between the controls, corresponding models, or vaccination groups (Supplementary Fig. 2a, b). Protein phosphatase 1 and regulatory subunit 1A (PPP1R1A), which is positively correlated with insulin impairment, were relatively stable in every group, except in one elder COVID-19 model in which these indexes were significantly elevated (599.9502 pg/mL) (Supplementary Fig. 2c). Amylase and lipase levels were measured to observe whether there was detectable pancreatitis in different experimental groups; there was no significant change compared with the corresponding control group (Supplementary Fig. 2d, e). Antibodies to the 65-kD isoform of glutamic acid decarboxylase (GAD65) are demonstrated to damage the structure and function of pancreatic β cells, which is positively associated with an increased risk of both type 1 and type 2 diabetes mellitus in adults.^28,29^ Strikingly, the level of GAD65 antibodies in the adult model was extremely significantly elevated compared with that in the adult control; furthermore, there was no significant change in the GAD65 level between the controls, corresponding models, or vaccination groups (Supplementary Fig. 2f). All these clinical data suggest that the β cell function was affected to a certain extent post-SARS-CoV-2 infection in adult models.

### Vaccination maintains homeostasis of insulin secretion by activating insulin receptors

We retrospectively collected pancreatic tissue samples from 188 rhesus macaque monkeys from different trials from 2020 to 2023. After evaluating the safety and efficacy of the vaccine, we selected 35 COVID-19 vaccines immunized and prototypic SARS-CoV-2 strain-infected adult NHPs models to compare with the uninfected adult control group and the prototypic SARS-CoV-2 strain-infected adult models group (Table 1) and further observed and studied the pancreatic tissues of animals in these groups.

For clinical data, there was no significant decrease in insulin secretion in the vaccination + COVID-19 model group (Supplementary Fig. 2b). Also, other serum parameters did not show an obvious difference between NHP models and vaccinated models (Supplementary Fig. 2a–f). To eliminate the effects of SARS-CoV-2 infection, the vaccine’s potential effect on pancreatic metabolism was directly observed by collecting and analyzing sera from 4 NHPs for 0–28 days post vaccination (dpv). Compared with fasting C-peptide and glucose levels and their ratio before vaccination, no significant changes in these levels were observed during the 28 dpv (Supplementary Fig. 2g). Importantly, glycated serum protein (GSP), which is a hemoglobin/erythrocyte-independent glycemic marker, reflects the average glucose concentration over the preceding 2–3 weeks.^30^ The GSP level was fairly stable during the 28 dpv (Supplementary Fig. 2h). The insulin levels showed an upward trend during the 4 weeks, and insulin expression significantly increased at 21 (*P* = 0.035) and 28 (*P* = 0.029) dpv, respectively, compared with those at 0 day (Supplementary Fig. 2i). Amylase levels also revealed an upward trend but without significant change compared with those at 0 day (Supplementary Fig. 2j). GAD65 expression increased transiently for 2–3 weeks after vaccination but without significant change compared with those at 0 day and returned to normal levels at 28 dpv (Supplementary Fig. 2k). ICAM-1 expression was slightly downward during the 4 weeks (Supplementary Fig. 2l).

The multi-label IF was examined to further explore in situ. Co-staining with trypsin; islet cell autoantibodies 512 (ICA512), which is an endocrine secretory granule marker; insulin receptor α (IRα); insulin receptor β (IRβ); and CK19, which is a cytoskeleton marker, was performed in the serial sections of pancreatic tissues in control, model, and vaccination + model groups (Fig. 3e–h). Firstly, SARS-CoV-2 infection partly elicits the expression of insulin receptor α, receptor β, and trypsin compared with the control group. Remarkably, ICA512 expression was significantly inhibited while IRα and IRβ expression significantly increased (Fig. 3i) in the vaccination + model group compared with both control and model groups, suggesting that although GAD65 might temporarily increase but back in the swing (Supplementary Fig. 2k), insulin receptors are activated to promote insulin absorption and function to maintain the homeostasis of insulin and glucose levels.

Collectively, these results indicate that vaccination maintains homeostasis of insulin secretion by activating insulin receptors and inhibiting ICA512 expression.

### Pathophysiologic mechanisms of SARS-CoV-2 infection causing islet impairment and loss of β cells in elder NHPs

We found mild pathological damage to the pancreas in COVID-19 models and that SARS-CoV-2 infection largely aggravates pathological phenotypes in elder NHPs. Here, we further explored the potential pathophysiological mechanism by which SARS-CoV-2 affects the pancreas, especially on islets. Firstly, markers of islet microcirculation damage, intercellular adhesion molecules (ICAM-1) and vascular cell adhesion molecule-1 (VCAM-1), expressed on inflamed vascular endothelium;^31^ Ras GTPase-activating protein-binding protein 1 (G3BP1), a marker of cell stress; and cleaved caspase 3, a marker of cell apoptosis, were detected and co-stained with glucagon and insulin protein (Fig. 4a, b). Specifically, compared with the elder control group, ICAM-1 and G3BP1 expression significantly increased in the elder model group, while VCAM-1 and cleaved caspase 3 expression had no significant change (Fig. 4c). Consistent with previous research, the percentage of glucagon^+^ insulin^+^ double cells was determined in the elder model group, suggesting SARS-CoV-2-induced β cell transdifferentiation^8^ (Fig. 4d). Furthermore, the percentage of ICAM^+^ β cells significantly increased, and that of G3BP1^+^ β cells significantly increased in the elder model group, but without significant differences in the percentage of VCAM-1^+^ β cells (Fig. 4e–g). These data suggest that pancreatic microcirculation, especially in the islets, was impaired to some degree and that islet β cells were exposed to an inflammatory environment and stress after SARS-CoV-2 infection in the elder NHPs.

**Fig. 6:**
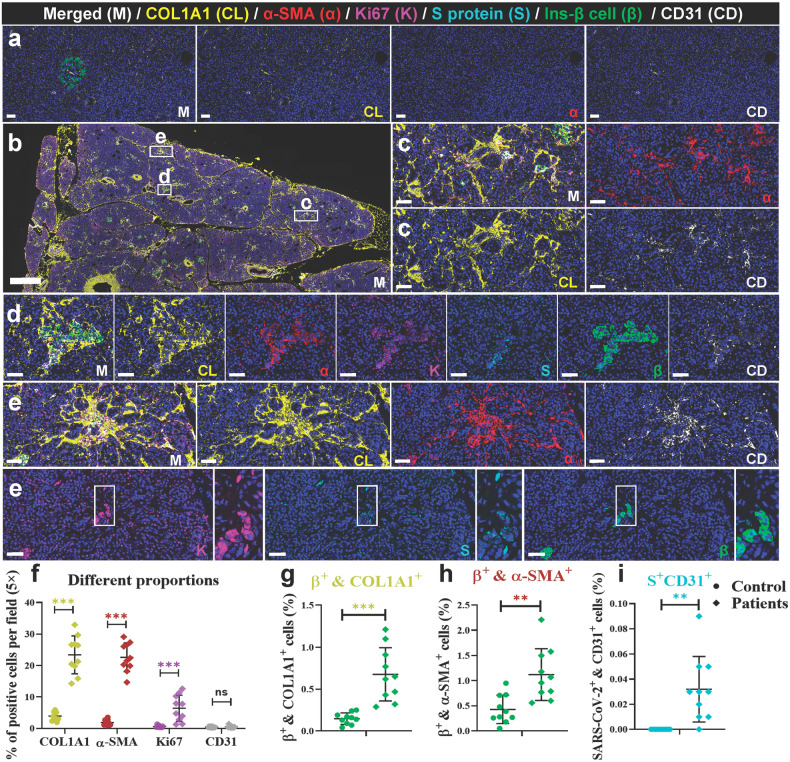
SARS-CoV-2 infection aggregated activation of α-SMA and accumulation of collagen fibers in the human pancreas. **a** Pancreatic tissue sections from deceased control subject were stained by multi-label immunofluorescence (IF) for COL1A1 (CL, yellow), α-SMA (α, red), Ki67 (K, magenta), SARS-CoV-2 S1 protein (S, cyan), insulin (β, green), and CD31 (CD, white). Scale bars, 800 μm. **b** Pancreatic tissue sections from the prototypic SARS-CoV-2 strain-infected COVID-19 autopsy samples were stained by multi-label immunofluorescence (IF) for COL1A1 (CL, yellow), α-SMA (α, red), Ki67 (K, magenta), SARS-CoV-2 S1 protein (S, cyan), insulin (β, green), and CD31 (CD, white). Scale bars, 800 μm. **c**–**e** Representative multi-label IF image from the magnified section of (**a**). Inset highlights activation of α-SMA and proliferation of collagen fibers in both the exocrine and endocrine pancreatic tissues. Scale bars, 50 μm. **f**–**i** Quantification of the percentage of COL1A1^+^, α-SMA^+^, Ki67^+^, and CD31^+^ cells, as well as insulin^+^COL1A1^+^, insulin^+^ α-SMA^+^, and S protein^+^ CD31^+^ cells, in the control human pancreatic tissues and the COVID-19 patients pancreatic tissues (*n* = 10 images/group). Data are presented as mean ± SD. p values were calculated by unpaired two-tailed Student’s *t* test. *p < 0.05, **p < 0.01, and ***p < 0.001

We observed focal lesions and suspected hyperplasia of collagen on HE slices in both the NHP model and autopsy samples. We further tested the expression of type 1 collagen (COL1A1); α-SMA, a marker of stellate cell activation; and CD31, a marker of vascular endothelium, and co-stained these marker proteins with Ki67, viral S protein, and insulin in NHP and human samples (Figs. 5 and 6).^32^ In the elder control group (Fig. 5a, b), both endocrine and exocrine regions of the pancreas showed minimal yellow-stained COL1A1+ collagen, primarily around blood vessels and connective tissues. These locations are typical for collagen fibers in normal histology. Furthermore, there is a scarce presence of activated α-SMA in the exocrine pancreas. Compared to the elder control group, two distinct pathological phenotypes were observed, depending on the location of collagen accumulation in the pancreas. In NHPs, SARS-CoV-2-infected elder NHPs (Fig. 5c–f) exhibited more clearly pathological phenotypes. In two animals from the elder model (2/6), proliferating collagen fibers distinctly divided the pancreas into different-sized lobules, and in some areas inside these lobules, the collagen fibers divided the pancreatic acini into different-sized islands, leading to loss of acinar cell mass. A few collagen fibers also accumulate in the islets. In the other type (4/6), islet amyloid tissues also robustly and extensively expressed COL1A1^+^ protein and remarkably replaced the islet cells, including β cells, leading to destruction of endocrine parenchyma and loss of β cells and eventually affecting endocrine metabolism, consistent with the results of Masson staining. Quantitatively, in the elder control group, the expression of COL1A1 was an average of 2.00%, the expression of α-SMA was an average of 4.59%, the expression of Ki67 was an average of 0.56%, and the expression of CD31 was an average 0.45%. Compared with the elder control group, the expression of COL1A1 (increased by 23.98%), α-SMA (increased by 14.97%), and CD31 (increased by 21.14%) significantly increased (Fig. 5h). Importantly, insulin^+^ COL1A1^+^ cells increased by 2.24% (Fig. 5i) and insulin^+^ α-SMA^+^ cells increased by 1.84% (Fig. 5j), compared with those of the elder control group, further suggesting an imbalanced expression and destruction of β cells. Consistent with our previous data, viral S protein^+^ CD31^+^ cells increased (Fig. 5k) and were extensively scattered in the severely impaired islets. Furthermore, the same panel of multi-label immunofluorescence was detected in the human samples, these pathological phenotypes were relatively slight. Compared with the control pancreatic tissues (Fig. 6a), there were also two pathological phenotypes in the COVID-19 patients’ pancreatic tissues (Fig. 6b). In the first type, collagen accumulated in and around the SARS-CoV-2-infected islet cells where stellate cells were mildly activated, and cell proliferation was slightly stimulated (Fig. 6c, d). In the second type, collagen that was extensively diffused and scattered in the interstitium of the exocrine pancreas and multi-focal tissue replaced the acinar cells. α-SMA, Ki67, and CD31 were also expressed (Fig. 6c, e). Quantitative analysis demonstrated that, compared with the control group, the expression of COL1A1 (increased by 19.52%), α-SMA (increased by 20.74%), and Ki67 (increased by 5.78%) significantly increased (Fig. 6f). Importantly, insulin+ COL1A1+ cells increased by 0.53% (Fig. 6g) and insulin+ α-SMA+ cells increased by 0.69% (Fig. 6h), compared with those of the control group. Consistently, the percentage of viral S protein^+^ CD31^+^ cells was 0.032 (Fig. 6i).

SARS-CoV-2 infection of elder NHPs’ pancreas initially damages the pancreatic microcirculation, increases the stress response of islet cells, expands the area of islet amyloidosis, and increases cytotoxicity. Along with the release of inflammatory and stress factors, stellate cells are activated, inducing a sharp increase in the extracellular matrix. Islet degeneration and necrosis stimulate the loss of β cells, directly inhibit insulin expression, and impair the expression of β cell-secreting proteins. Finally, the number of β cells progressively decreased, suggesting that SARS-CoV-2 might aggravate the geriatric development of a diabetes-mellitus-like pathological phenotype in elder NHPs.

### Proteomics, lipidomics, and metabolomics display differential metabolic characteristics among SARS-CoV-2-infected adult, elder, and vaccinated NHPs, consistent with their pathological phenotype

Omics-based technologies, including proteomics, lipidomics, and metabolomics, have been adopted during the COVID-19 pandemic to understand the pathophysiological processes and biochemical mechanisms behind infection and spread.^33^ Here, we examined, analyzed, and compared the sera before and after SARS-CoV-2 infection or vaccination from NHPs with combined multi-omics to explore the potential relationship between pancreatic and metabolic alterations.

A total of 27 serum samples were collected, including the following: 3 serum samples from control NHPs, 5 samples from aged monkeys 3 days post-infection with prototypic SARS-CoV-2 strain, 5 samples from aged monkeys 7 days post-infection with prototypic SARS-CoV-2 strain, 3 samples from adult monkeys 7 days post-infection with prototypic SARS-CoV-2 strain, 4 adult samples 7 days post-infection with delta strain, 3 samples from adult monkeys infected by the prototypic SARS-CoV-2 strain after being vaccinated twice, and 4 samples from adult monkeys infected by the delta strain after being vaccinated twice.

Firstly, through quantitative proteomics, metabolomics, and lipidomics analysis, we sorted out the total differential metabolite numbers among different groups. To be clear, metabolites upregulated or downregulated in serum were marked with different colors within the groups. Overall, compared with the control group, we evaluated different model groups; remarkably, the aged animals infected with SARS-CoV-2 for 7 days had more differentially expressed metabolites (440 in total). Compared with the adult-infected model, the aged-infected model had the highest total of differentially expressed metabolites (i.e., 580) (Supplementary Fig. 3a). This is consistent with the trend of pathological phenotypes. Furthermore, the difference between the vaccinated-prototypic SARS-CoV-2 strain-infected group and the control group was large (562 in total). Venn of proteomics, metabolomics, and lipidomics analysis between the control and different model groups, as well as among the control group, different model groups, and vaccinated-infected groups, is shown in Supplementary Fig. 4b, c. Although the amounts of differential metabolites were found between the control group and other groups, the same differential metabolites among different groups were relatively few, even without the intersection between the vaccination-related groups (Supplementary Fig. 3c). The differential metabolites screened out among the vaccination-related groups in the metabolomics analysis included 2-hydroxy-2-methylbutanenitrile, 4-pyridoxic acid, astaxanthin, dihydroactinidiolide, LPC(20:5/0:0), LPC(O-20:2), propylparaben, and δ-valerolactam. The differential metabolites screened out among the vaccination-related groups in the lipidomics analysis included lysophosphatidylcholine (LPC(20:5/0:0) and LPC(24:1/0:0)); phosphatidylcholine (PC(14:0_20:5), PC(14:0_22:6), PC(16:1_22:6), PC(18:2_20:5), PC(O-14:0_20:4)); and phosphatidylinositol (PI(16:0_20:5)), whose signal transduction pathway is related to the regulation of glucose metabolism.^34^

These data suggest that SARS-CoV-2 infection affects host metabolism, especially glucose and lipid metabolism. Along with age and a longer duration of infection, adverse effects gradually increase. Additionally, the metabolic effects of the vaccine on infected animals were mild.

### Multi-omics analysis highlights insulin-resistance-related metabolite enrichment in the adult COVID-19 model and aggregated-pancreatic-lesion-related metabolic dysfunction in the elder model

**Fig. 7:**
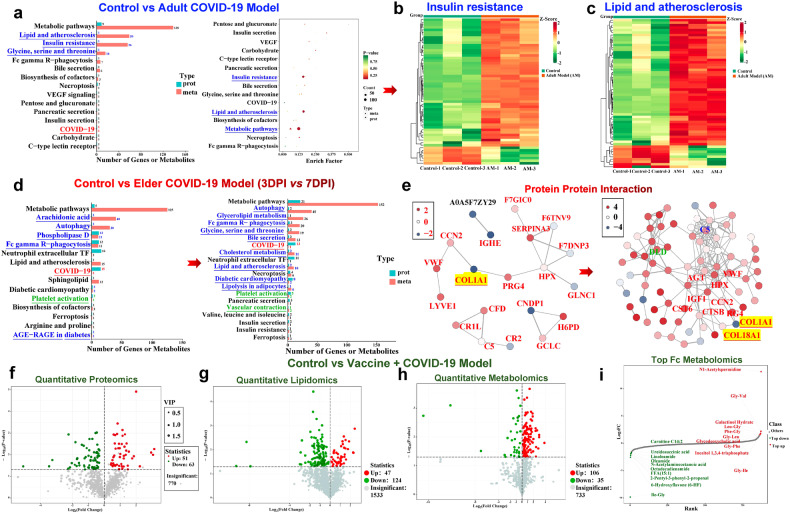
SARS-CoV-2 infection triggers distinct characteristics of proteomics and metabolomics among adult, elder, and vaccinated COVID-19 NHP models. **a** Bar and dot plots of the KEGG pathway combined functional analysis of the enriched pathways associated with differentially expressed proteins and metabolites between the control and adult prototypic SARS-CoV-2 strain-infected model groups. **b**, **c** Heatmap revealing that genes corresponding to differential proteins and metabolites are mainly enriched in the insulin resistance pathway and lipid and atherosclerosis pathway. **d** Bar plots of the KEGG pathway combined functional analysis of the enriched pathways associated with differentially expressed proteins and metabolites from 3 days post-infection (DPI) to 7 DPI from the elder prototypic SARS-CoV-2 strain-infected COVID-19 model compared with those of the control group. In **a** and **d**, the blue characters represent the signaling pathway directly related to glycometabolism involved in the islets, the red characters represent the metabolic pathway directly related to COVID-19, and the green characters represent the metabolic pathway related to blood circulation. **e** Correlation networks of significant protein interaction. Each node in the interaction network represents a protein, and the change in node color from red to blue represents the change in protein expression level from being upregulated to being downregulated. The thickness of the lines represents the change from high to low confidence in interaction relationships. **f** Volcano plot showing the relative content differences of proteins between the control and the prototypic SARS-CoV-2 strain-infected model vaccinated with inactivated vaccines and the significance of statistical differences. Each dot in the volcano plot represents a protein, with green dots representing downregulated differential proteins, red dots representing upregulated differential proteins, and gray dots representing proteins detected but not significantly different. **g**, **h** Volcano plot showing the relative content differences of lipids and metabolites between the control and vaccinated COVID-19 model groups and the significance of statistical differences, respectively. Each dot in the volcano plot represents a lipid or a metabolite, with green dots representing downregulated differential lipids or metabolites, red dots representing upregulated differential lipids or metabolites, and gray dots representing lipids or metabolites detected but not significantly different. **i** Dynamic distribution of metabolites. The abscissa represents the cumulative number of substances in the order of difference multiple from small to large, and the ordinate represents the pair value with the difference multiple as base 2. Each dot represents a substance, the green dot represents the substance that is in the top 10 ranking down, and the red dot represents the substance that is in the top 10 ranking up. The KEGG pathway name with blue characters represents significantly enriched pathways related to diabetes mellitus and glucose metabolism impairment. The KEGG pathway name with red characters represents a SARS-CoV-2-associated pathway. The KEGG pathway name with green characters represents significantly enriched pathways related to thrombogenesis

Proteomics, lipidomics, and metabolomics combined with multi-omics analysis were used to explore the potential relationship between SARS-CoV-2-infection-induced islet impairment and metabolic status, as well as the relationship with age and duration of infection. Through multi-omics analysis, we found that compared with the control group, the genes corresponding to differential proteins and differential metabolites were mainly enriched in the insulin resistance pathway (1 gene and 56 metabolites) and lipid and atherosclerosis pathway (1 gene and 59 metabolites). Moreover, the glycine, serine, and threonine metabolic pathways (1 gene and 16 metabolites) were also enriched. Previous research has demonstrated that glycine and serine interact with NMDA glutamate receptors and may regulate insulin secretion in β cells.^35^ SARS-CoV-2 infection also affects cell-metabolism-related pathways, such as Fc-gamma-R-mediated phagocytosis and necroptosis; pathways related to exocrine and endocrine gland secretion such as bile, pancreatic, and insulin secretion; and pathways related to glucose metabolisms such as carbohydrate digestion and absorption (Fig. 7a). The genes and metabolites that were enriched in the insulin resistance pathway and lipid and atherosclerosis pathway are illustrated in Fig. 7b, c, respectively.

Using multi-omics analysis, we then compared the control group with the elder model that was infected for 3 or 7 days. From the gene or metabolite enrichment in the multi-omics analysis, we found that, along with a prolonged infection time, the number of differential metabolites sharply increased, and the pathway number of metabolic dysfunction and corresponding differential genes or metabolites increased. Metabolic disorders progressively worsened (Fig. 7d). Importantly, correlation networks of differential metabolites are shown in Fig. 7e. We observed that the serum COL1A1 level extremely decreased from 3 dpi to 7 dpi. Furthermore, COL1A1-related differential metabolites increased, suggesting that COL1A1 reduction affects a cascade of reactions to aggravate metabolic dysfunction. Increased collagen type XVIII alpha 1 chain (COL18A1) may be related to altered COL1A1. Specifically, at 3 dpi, 75 differential proteins between the control and 3-dpi groups and proteomics alterations are summarized by heatmap comparison in Supplementary Fig. 4a. There were 30 downregulated proteins and 45 upregulated proteins summarized in the volcano map (Supplementary Fig. 4b). Some differential proteins were enriched in extracellular structures and posttranslational modification, and protein turnover chaperone pathways, suggesting its relationship with islet amyloidosis. Some differential proteins were enriched in carbohydrate transport and metabolism, and energy production and conversion, suggesting glucose metabolism dysfunction (Supplementary Fig. 4c). The analysis of Gene Ontology (GO) in cellular components (CCs) consistently demonstrated that differential genes were enriched in the extracellular region (Supplementary Fig. 4d). Additionally, of the 83 differential metabolites between the control and 3-dpi groups, there were 39 downregulated proteins and 44 upregulated proteins summarized in the volcano map (Supplementary Fig. 4e). Of the 240 differential lipids between the control and 3-dpi groups, there were 194 downregulated proteins and 46 upregulated proteins summarized in the volcano map (Supplementary Fig. 4f). Of the 125 differential proteins between the control and 7-dpi groups and proteomics alterations summarized by heatmap comparison in Supplementary Fig. 5a, there were 23 downregulated proteins and 102 upregulated proteins summarized in the volcano map (Supplementary Fig. 5b). KEGG pathways showed that some proteins were enriched in the glycosaminoglycan degradation, glyoxylate and dicarboxylate metabolism, diabetic cardiomyopathy, insulin secretion, and glycolysis/gluconeogenesis pathways, which are all associated with diabetes-related glucose metabolic disorders. Dysfunction in tryptophan and beta-alanine metabolism might alter insulin signaling in cells through the mTOR pathway (Supplementary Fig. 5c).^36^ Furthermore, through electron microscopy, we observed damage to cellular organelles in the islet cells, myeloid degeneration, and mitochondrial swelling in the infected elder animals (Supplementary Fig. 5d). Progressively, the functions of differential proteins were almost enriched in glucose- and lipid-metabolism-related pathways including energy production and conversion, extracellular structures, amino acid transport and metabolism, carbohydrate transport and metabolism, post-translational modification, protein turnover, chaperones, intracellular trafficking, secretion, and vesicular transport (Supplementary Fig. 5e). Of the 128 differential metabolites between the control and 7-dpi groups and metabolic alterations summarized by heatmap comparison in Supplementary Fig. 5f, there were 44 downregulated metabolites and 84 upregulated metabolites summarized in the volcano map (Supplementary Fig. 5g). Bar charts of differential metabolites for the top 20 upregulated and downregulated metabolites are summarized in Supplementary Fig. 5h. Of the 297 differential lipids between the control and 7-dpi groups, there were 228 downregulated lipids and 69 upregulated lipids summarized in the volcano map (Supplementary Fig. 5i). The dynamic distributions of differential lipids for the top 10 upregulated and top 10 downregulated lipids are summarized in Supplementary Fig. 5j.

Finally, vaccine-related metabolic alterations were analyzed. Of the 114 differential proteins between the control and vaccinated COVID-19 model groups, there were 63 downregulated proteins and 51 upregulated proteins summarized in the volcano map (Fig. 7f). Of the 171 differential lipids between the control and vaccinated COVID-19 model groups, there were 124 downregulated and 47 upregulated lipids summarized in the volcano map (Fig. 7g). Of the 141 differential metabolites between the control and vaccinated COVID-19 model groups, there were 35 downregulated and 106 upregulated metabolites summarized in the volcano map (Fig. 7h). The dynamic distributions of differential lipids and metabolites for the top 10 upregulated and downregulated lipids and metabolites are summarized in Fig. 7i, respectively. We observed that PE (O-20:0_20:5), CE (23:0), and N1-acetylspermidine significantly increased, which may be potential biomarkers worthy of further exploration.

## Discussion

Our study revealed that SARS-CoV-2 infection can lead to various levels of pancreatic impairment and glycometabolic dysfunction, especially in elder NHP models. SARS-CoV-2 infection negatively affects glucose metabolism in adult rhesus monkeys even before the manifestation of pathological phenotypes, while it extensively exacerbates multiple pathological transformations in elder models. Conversely, COVID-19 vaccination maintains homeostasis of insulin secretion by activating insulin receptors.

In line with previous reports,^10,11,37^, our findings demonstrate that SARS-CoV-2 can directly infect nearly all types of exocrine and endocrine pancreatic cells in both human and NHP models and then adversely affect the pancreas. Besides focal inflammation and degeneration, another affected parameter suggestive of pancreatic dysfunction in adult NHP models is GAD65, which is identified as a major autoantigen in insulin-dependent diabetes.^29^ High titers of GAD65 and autoantibodies against GAD65 (GAD65Ab) have also been detected in other autoimmune diseases and type 2 diabetes.^28,38–40^ In the pancreas, GAD65 is predominantly expressed in pancreatic β cells under physiological conditions, responsible for catalyzing the production of gamma-aminobutyric acid from l-glutamic acid to main normal insulin secretion and the GAD65 level in serum or plasma is relatively low. However, GAD65 will be released into the blood when the islet β cells are damaged, which can occur in autoimmune diseases (such as type 1 diabetes) or other disorders such as pancreatitis. Hence, although GAD65Ab tests are more commonly used, increased GAD65 levels can also reflect functional impairment of islet β cells to a certain extent.^41^ Notwithstanding, the damage to endocrine cells did not seem severe enough to induce hyperglycemia or diabetes in adult model monkeys. These observations align with other studies showing that only a minority of patients with acute COVID-19 exhibit signs of β cell failure and diabetes.^42–44^

As indicated by multi-omics data and increased levels of fasting C-peptide and C-peptide/glucose, SARS-CoV-2 infection results in insulin resistance and glycometabolic dysfunction in adult models.^45^ This phenomenon is also observed in some individuals post-COVID-19 infection, who reported increased hunger despite normal levels of blood glucose and insulin, but with elevated C-peptide levels.^46,47^ Through post hoc analysis, Clarke et al. reported higher C-peptide levels were higher in survivors of COVID-19 with severe or critical disease compared to those with mild disease, suggesting a potential link between C-peptide and COVID-19 disease severity.^48^ However, the cause of C-peptide elevation warrants further investigation. Given the normal insulin secretion, the elevated C-peptide might be due to reduced clearance by the kidneys, which could be impaired owing to SARS-CoV-2 infection.^49,50^

Regarding insulin resistance in adult NHP models, it appears to be minimally associated with the limited pathological injury of the pancreas. It is well established that insulin resistance, particularly diminished insulin responses in peripheral tissues such as skeletal muscle, adipose tissue, and liver, in combination with or without β cell dysfunction, ultimately leads to hyperglycemia and a series of complications.^51^ Recently, several studies have investigated the mechanisms of COVID-19-related insulin resistance. Reiterer et al. reported that insulin resistance is the primary cause of hyperglycemia in patients with severe COVID-19. They found that SARS-CoV-2 can directly infect human and mouse adipocytes, triggering an inflammatory antiviral response in the adipose tissue and driving insulin resistance in acute COVID-19.^42^ Another study identified replicating viruses in human hepatocytes from postmortem liver biopsies and in primary hepatocytes, which can abate these cells’ sensitivity to insulin and induce glucose production through gluconeogenesis.^52^ Conjointly, these findings strongly suggest that COVID-19-related insulin resistance is probably on account of the impaired peripheral tissues regardless of pancreas and β cell function.

Generally, the pathological damage to the pancreas in older models was more severe than in adult models. This was evident not only in the increased proportion of animals with lesions but also in the heightened severity of lesions in affected animals with pancreatic impairment (Fig. 3). Simultaneously, metabolic pathway disorders, such as glycolipid metabolism, were exacerbated, particularly in elder infected monkeys at 7dpi (Fig. 7 and Supplementary Figs. 3–5). In harmony with previous reports,^53^ of our molecular pathology results and multi-omics data suggest that SARS-CoV-2 infection intensifies pancreatic lesions and heightens the risk of diabetes in the elderly.

As discussed in the context of adult models, the increased risk of insulin-resistant diabetes in the elder model is supported by the following three points: (1) multi-omics data, which includes multiple altered proteins and metabolites implicated in insulin-resistant (Fig. 7d, e, Supplementary Figs. 4 and 5); (2) pathological phenotypes reminiscent of Type 2 diabetes, such as inflammation and amyloid deposition; and (3) corroborating results from other research groups.^42,52^ Amyloid deposition within islets in Type 2 diabetes typically commences in and around the capillaries and between cells. In advanced stages, islets can be obliterated, and fibrosis may be observed.^54^ Here, we also observed amyloid deposition and excessive collagen fiber formation, akin to Type 2 diabetes.^55,56^ However, it’s worth noting that amyloid deposition and fibrosis may also be found in older non-diabetic individuals, suggesting that SARS-CoV-2 infection may exacerbate the microscopic tissue changes associated with normal aging.

In considering the heightened risk of insulin-dependent diabetes in the elderly following COVID-19, our attention is drawn to the β cells. Based on our pathophysiological findings, we posit that SARS-CoV-2 infection may harm β cells in older models via several mechanisms: (1) SARS-CoV-2 directly infects β cells, leading to cellular degeneration and necrosis, a finding consistent with previous studies;^9,10^ (2) SARS-CoV-2 infects and damages pancreatic endothelial cells, thereby increasing their permeability.^57^ This not only facilitates viral entry into the pancreas but also enables the exudation and infiltration of inflammatory cells and cytokines into the pancreas, triggering a stress response in pancreatic cells and impairing β cells; (3) Amyloid deposition in the islets constricts the space of intrinsic islet cells and alters the β cell microenvironment;^58^ (4) Activated stellate cells promote collagen fibers formation, leading to changes in the islet microenvironment.^56^ All these factors can induce β cell degeneration and necrosis, subsequently reducing in situ insulin secretion. Contrary to existing literature, we did not detect apoptosis in the islets, which may be due to differences in the models or methods used.^10,43,59,60^ Here, we observe the tendency of reduction in serum insulin levels and a tendency of enhancement in serum glucose in the elder monkey models compared to the elder controls but without significant difference, which might be due to small sizes in these groups. We observed a significant decrease in the expression of insulin and β cell damage in situ in the pancreatic section, consistent with the typical histological damage in clinical diabetes patients. Overall, we propose that SARS-CoV-2 infection may aggravate the geriatric development of diabetes-like pathological phenotypes in elder NHPs.

Due to the retrospective nature of our experiment based on samples collected between 2020 and 2023, there was a limited availability of serum samples, which precluded the assurance of sufficient sample sizes across all groups. However, we ensured the quality of all utilized samples and incorporated considerations for these variations in sample volume into our multi-omics analytical methods and statistical approaches. The rhesus macaque model samples we collected were from infections lasting seven days, primarily investigating the short-term impacts of COVID-19 infection on pancreatic damage. However, the effects of long-term or recurrent infections on the pancreas remain unclear. Future studies could delve deeper into animal models of long COVID or recurrent COVID-19 infections. This study validates the findings observed in the rhesus macaque model with pancreatic samples from clinical COVID-19 patients, further confirming the occurrence of pancreatic damage and stellate cell activation in COVID-19 patients.

Our study’s strength lies in the retrospective analysis of the potential relationship between SARS-CoV-2 and host pancreatic damage and glycemic alterations, utilizing multiple methodologies. We conducted qualitative and quantitative analyses comparing the control and experimental groups across human and rhesus monkey samples, and the results showed a remarkable consistency. The observed pancreatic damage in COVID-19 patients and infected rhesus macaques, particularly more pronounced in older individuals, suggests that in the treatment of COVID-19 patients, attention should not only be given to respiratory inflammation but also to changes in clinical indicators related to glycemia and pancreatic damage, especially in the elderly. These findings align with clinical data and present preliminary insights into metabolic issues based on pancreatic pathology, offering valuable foundational data for clinical research.

In conclusion, we report different effects of SARS-CoV-2 infection and COVID-19 vaccines on the pancreas. Our results suggest that the pancreatic impairment caused by SARS-CoV-2 infection is age-related and that vaccination maintains homeostasis of insulin secretion by activating insulin receptors. We hope these findings will provide insights into the occurrence of disorders related to pancreatic injury following COVID-19.

## Materials and methods

Key resources table

**Table:** 

Reagent or resource	Source	Identifier
Antibodies		
Anti-SARS spike glycoprotein antibody [1A9]	Abcam	Cat# ab273433
GeneTex:SARS-CoV-2 (COVID-19) nucleocapsid antibody [HL5511]	GeneTex	Cat# GTX635689
SARS-CoV-2 (COVID-19) nucleocapsid antibody [HL455-MS]	GeneTex	Cat# GTX635712
SARS-CoV-2 (COVID-19) Spike S1 antibody [HL6]	GeneTex	Cat# GTX635654
Anti-Ribonuclease 3/ECP antibody	Abcam	Cat# ab207429
Anti-Insulin antibody [EPR17359]	Abcam	Cat# ab181547
Glucagon Antibody	Cell Signaling Technology	Cat# 2760
Anti-Somatostatin 28 antibody [EPR3359(2)]	Abcam	Cat# ab111912
Anti-Pancreatic Polypeptide Antibody [EPR23320-10]	Abcam	Cat# ab272732
Anti-Insulin Receptor alpha antibody [EPR23962-157]	Abcam	Cat# ab283689
Anti-Insulin Receptor beta antibody [EPR23566-103]	Abcam	Cat# ab278100
Anti-Trypsin antibody [EPR19497]	Abcam	Cat# ab200996
Anti-ICA 512/PTPRN antibody [EPR20718]	Abcam	Cat# ab207750
Anti-Cytokeratin 19 antibody [EP1580Y] - Cytoskeleton Marker	Abcam	Cat# ab52625
Anti-ACE2 antibody	Abcam	Cat# ab15348
Anti-TMPRSS2 antibody [EPR3862]	Abcam	Cat# ab109131
Anti-Neuropilin 1 antibody [EPR3113]	Abcam	Cat# ab81321
CD54/ICAM-1 (E3Q9N) XP	Cell Signaling Technology	Cat# 67836 S
Anti-VCAM1antibody [EPR5047]	Abcam	Cat# ab134047
Cleaved Caspase-3 (Asp175) Antibody	Cell Signaling Technology	Cat# 9661
Anti-G3BP1 antibody	Cell Signaling Technology	Cat# 17798
Anti-alpha smooth muscle Actin antibody [SP171]	Abcam	Cat# ab150301
COL1A1 (E8F4L) XP® Rabbit mAb	Cell Signaling Technology	Cat# 72026
Anti-Ki67 antibody	Abcam	Cat# ab15580
Anti-CD31 antibody	Abcam	Cat# ab28364
Anti-Myeloperoxidase antibody [EPR20257]	Abcam	Cat# ab208670
Anti-CD4 antibody [UMAB64]	Zsbio	Cat# ZA-0519
Anti-CD20 antibody [EP459Y]	Abcam	Cat# ab78237
Anti-CD68 antibody [C68/684]	Abcam	Cat# ab201340
Critical commercial assays		
Monkey Insulin ELISA Kit (Colorimetric)	NOVUS	Cat# NBP2-60076; RRID: C1482161422
Cynomolgus Monkey C-Peptide ELISA Kit (Colorimetric)	NOVUS	Cat# NBP2-59957; RRID: C1482031409
Amylase Assay Kit (Colorimetric)	Abcam	Cat# ab102523; RRID: 1010078-3
Monkey LIPF/GL/Gastric Lipase (Sandwich ELISA) ELISA Kit - LS-F45315	Lifespan	Cat# LS-F45315-1; RRID: 224374
Glucose Assay Kit (Colorimetric/Fluorometric)	NOVUS	Cat# KA0831
Monkey GAD65 (Sandwich ELISA) ELISA Kit - LS-F44382	Lifespan	Cat# LS-F44382-1; RRID: 224373
Monkey ICAM-1/CD54 ELISA Kit (Colorimetric)	NOVUS	Cat# NBP1-92712; RRID: 325366-005
Monkey Vascular Cell Adhesion Molecule 1 (VCAM1) ELISA Kit	Abbexa	Cat# abx153571; RRID: E2212721G
Porcine PPP1R1A / IPP1 (Sandwich ELISA) ELISA Kit - LS-F47439	Lifespan	Cat# LS-F47439-1; RRID: 224375
Bond Polymer Refine Detection	Leica Biosystems Newcastle Ltd.	Cat# DS9800-CN
Bond Wash Solution 10X Concentrate	Leica Biosystems Newcastle Ltd.	Cat# AR9590-CN
Bond Dewax Solution	Leica Biosystems Newcastle Ltd.	Cat# AR9222-CN
Bond Epitope Retrieval Solution 1	Leica Biosystems Newcastle Ltd.	Cat# AR9961-CN
Bond Epitope Retrieval Solution 2	Leica Biosystems Newcastle Ltd.	Cat# AR9640-CN
Bond Primary Antibody Diluent	Leica Biosystems Newcastle Ltd.	Cat# AR9352-CN
Bond Research Detection System	Leica Biosystems Newcastle Ltd.	Cat# DS9455
Bond Aspirating Probe Cleaning Kit	Leica Biosystems Newcastle Ltd.	Cat# CS9100
Bond Open Container 7 mL - 10 Pack	Leica Biosystems Newcastle Ltd.	Cat# OP79193
Bond Titration kit - 10 Pack	Leica Biosystems Newcastle Ltd.	Cat# OPT9049
Opal Polaris 7-Color Manual IHC Kit	Akoya Biosciences	Cat# NEL861001KT
OPAL 7-Color auomation IHC KIT 50 SLIDES	Akoya Biosciences	Cat# NEL821001KT
Opal Polaris 480 Reagent Pack	Akoya Biosciences	Cat# FP1500001KT
Opal Polaris 780 Reagent Pack	Akoya Biosciences	Cat# FP1501001KT
RNAscope Probe-V-nCoV2019-S	Advanced Cell Diagnostics	Cat# 848561
2.5HD DAB detection kit	Advanced Cell Diagnostics	Cat# 322300
Modified Masson’s Trichrome Stain Kit	Beijing Solarbio Science & Technology Co., Ltd.	Cat# G1346; RRID: 20220511
Congo Red Stain Kit	Abcam	Cat# ab150663; RRID: GR3330147-1
Eosinophil-Mast Cell Stain Kit	Abcam	Cat# ab150665
Software and algorithms		
ImageJ 1.53c	NIH	https://imagej.nih.gov/ij/
GraphPad Prism 8.0	GraphPad Software	https://www.graphpad.com
HALO v3.0.311.262	Indica Labs	https://indicalab.com/halo/
PerkinElmer’s inForm® advanced image analysis software	PerkinElmer, Inc.	https://www.perkinelmer.com.cn/
Phenochart 1.1.0	PerkinElmer, Inc.	https://www.perkinelmer.com.cn/

### Ethics statement and animal experiments

In this study, human pancreatic specimens are used for comparative medical research. The pancreatic tissue slices from two post-mortem COVID-19 patients were a gift from Prof. Xiuwu Bian. These samples are from the study which was approved by the ethics committee of Huoshenshan Hospital (KY2020298).^61^ The detailed information of these two patients (cases 1 and 2) was described as before,^61^ whose survival time since symptom onset was 65 and 29 days, respectively. The control of human pancreas samples was approved by the Institutional Review Board of the Institute of Basic Medical Sciences, Chinese Academy of Medical Sciences (Approval Number: 009-2014, 2022125). Sample ID: 818, derived from a donor aged 85 with no history of cancer or diabetes. The donor had a longstanding history of hypertension and had experienced multiple cerebrovascular accidents. Tissue procurement was conducted with a post-mortem delay of 4 h, ensuring minimal degradation of biological markers. For NHPs, all animal experiments were performed in an animal biosafety level 3 (ABSL3) facility with high-efficiency particulate air (HEPA)-filtered isolators. All research was performed in compliance with the Animal Welfare Act and other regulations relating to animals and experiments. In this retrospective study, adult rhesus macaques (Macaca mulatta) were 3–5 years old, and elder rhesus macaques were 15–35 years old (the date of birth in months/date/years of elder animals were listed in the following table). These age ranges align with the typical lifespan and aging process of NHPs and are indicative of adult and elderly stages in human terms. The Institutional Animal Care and Use Committee of the Institute of Laboratory Animal Science (ILAS), Peking Union Medical College (PUMC), reviewed and authorized all protocols in this research, including research performed in animals. Briefly, all the samples were collected after anesthetized with 10 mg/kg ketamine hydrochloride.^16,23,24,62–64^

**Table:** 

Animal no.	Date of birth	Date of dissection	Age at death
Elder model 1	06/08/1997	09/10/2020	23
Elder model 2	0713/1989	09/10/2020	31
Elder model 3	08/03/1995	09/10/2020	25
Elder model 4	07/23/1998	09/11/2020	22
Elder model 5	01/09/1996	09/11/2020	24
Elder model 6	06/03/1996	09/11/2020	24
Elder control 1	05/07/1995	09/12/2020	25
Elder control 2	05/03/1994	09/12/2020	26
Elder control 3	08/03/1994	09/12/2020	26

## Method details

All studies were performed in a blinded manner without inclusion and exclusion applied. The sample size and statistical analysis method of each experiment have been provided in the figure legends.

### Virus

The SARS-CoV-2 virus is designated as SARS-CoV-2/WH-09/human/2020/CHN (GenBank: MT093631.2), SARS-CoV-2/human/CHN/Delta-1/2021 (GenBank: OM061695.1) and SARS-CoV-2/human/CHN/Omicron-1/2021 (Genbank: OM095411.1) were provided by ILAS, PUMC, China. To identify the stocks of the virus, the plaque-purified viral isolate was amplified as described previously.^23^

### Cells

Vero cells were used for the reproduction of SARS-CoV-2 stocks. Vero cells are maintained in Dulbecco’s modified Eagle’s medium (DMEM, Invitrogen, Carlsbad, CA, USA) supplemented with 10% fetal bovine serum, 100 IU/mL penicillin, and 100 µg/mL streptomycin, and incubated at 37 °C, 5% CO_2_. Titers for SARS-CoV-2 were resolved by a 50% tissue-culture infectious doses (TCID_50_) assay.^16^

### Hematoxylin and eosin staining

All collected organs were fixed in 10% buffered formalin solution in a wet specimen bank, and paraffin sections (3–4 µm in thickness) were prepared according to routine practice. All tissue sections were stained with H&E. Histopathological changes in different tissues were observed using an Olympus microscope.^62^

### Histopathology and immunohistochemistry (IHC)

All collected organs were fixed in a 10% buffered formalin solution, and paraffin sections (3–4 µm in thickness) were prepared as described in a previous report.^62^ We have tested and validated different commercial antibodies to confirm if the positive signal of viral antigens or different antibodies are specific reaction. Briefly, paraffin sections (3–4 μm in thickness) were prepared and stained with H&E prior to observation by light microscopy. For IHC staining or preliminary experiments to identify the expression of different antibodies by manual operation, dehydrated paraffin sections (3–4 µm in thickness) were treated with an antigen retrieval kit (AR0022; Boster Bio, Pleasanton, CA) for 1 min at 37 °C and quenched for endogenous peroxidases in 3% H_2_O_2_ in methanol for 10 min. After blocking in 1% normal goat serum for 1 h at room temperature, the sections were stained with different antibodies at 4 °C overnight, followed by incubation with horseradish peroxidase (HRP)-labeled goat anti-mouse IgG secondary antibody (ZDR-5307, 1:200; ZSGB Bio), HRP-labeled goat anti-rabbit IgG secondary antibody (ZDR-5306, 1:200; ZSGB Bio), or HRP-labeled goat-anti rat IgG secondary antibody (ZF-0312, 1:200; ZSGB Bio) for 1 h. Finally, the sections were visualized by incubation with 3,3′-diaminobenzidine tetrahydrochloride (DAB) and viewed using an Olympus microscope. Sequential sections from all collected tissues were directly incubated with HRP-labeled goat anti-mouse or anti-rabbit IgG and used as the omission control for different antibody staining. Sequential sections from all collected tissues were incubated with a recombinant anti-rabbit IgG antibody [SP137] (ab208334, 1:1000; Abcam) as the negative control for protein expression.

### Masson’s trichrome staining, congo red staining, and eosinophil-mast cell staining

Serial sections (3–4 μm in thickness) were prepared and stained with Modified Masson’s Trichrome Stain Kit, Congo Red Stain Kit, and Eosinophil-Mast Cell Stain Kit according to the manufacturer’s instructions.

### IHC optimization

IHC optimization was performed using an automated staining system, Leica BOND RXm stainer (Leica Biosystems), with previously optimized and validated antibodies listed in the Key resources table. In total, 3–4 μm formalin-fixed paraffin-embedded tissue sections were baked and dewaxed (Dewax Solution, Leica). Antigen retrieval and antibody stripping steps were performed at 100 °C using Epitope Retrieval Solution 1 or Epitope Retrieval Solution 2, depending on the datasheet of antibodies and Bond Wash Solution (Leica). Expression of all cell markers was detected using a Novocastra BOND Polymer Refine Detection Kit, with a diaminobenzidine reaction to detect antibody labeling and hematoxylin counterstaining. To obtain uniform staining, several tests were performed using different antibody dilutions and antigen retrieval conditions until optimal conditions were obtained for the primary and secondary antibodies in the positive controls.^24^

### Multiplex immunofluorescence staining and confocal microscopy

We tested and validated different commercial antibodies to confirm if the positive signals a specific reaction. Multiplex immunofluorescence staining was performed using an Opal Polaris 7-Color manual IHC Kit or Opal 7-Color automation IHC Kit. Different primary antibodies were sequentially applied to examine specific cell markers, followed by a fluorophore from the Opal 7 color IHC kit, including DAPI and Opal Polaris 520, 540, 570, 620, 650, and 690. The color corresponds to each cell marker/receptor/ targeted protein in different figures shown in the following table. For the new panels with seven antibodies, the TSA fluorophore Opal Polaris 480 (#FP1500001KT, Akoya Biosciences) was added to the kit. HRP-conjugated secondary antibody incubation and tyramide signal amplification (TSA). The slides were microwave-treated after each cycle of TSA. Nuclei were stained with 4’-6’-diamidino-2-phenylindole (DAPI; Sigma-Aldrich, St. Louis, MO) after antigen labeling for 10 min and cover-slipped with Prolong Gold Antifade. Stained slides were scanned using the Leica Application Suite X (Leica Microsystems, Wetzlar, Germany), which captures fluorescent spectra at 20-nm wavelength intervals from 420 to 720 nm with identical exposure times, and the scans were combined to build a single stacked image; or were scanned using the Akoya Vectra Polaris state-of-the-art multispectral imaging system, which enables the detection and downstream quantification of multiple overlapping biomarkers as the signals are unmixed from one another and from autofluorescence. Images were spectrally unmixed using PerkinElmer inForm software, exported as multi-image TIFF files, and analyzed using Phenochart. The bars and colors of the bars were generated automatically by Phenochart.

**Table:** 

Panels/colors	Green	Red	Cyan	Magenta	Yellow	White
Panel 1 in Fig. 1	SARS-CoV-2 S1 protein	Glucagon^+^ α cell	Insulin^+^ β cell	Somatostatin^+^ δ cell	Pancreatic polypeptide^+^ PP cell	
Panel 2 in Fig. 2	SARS-CoV-2 S1 protein	Glucagon^+^ α cell	Insulin^+^ β cell		Somatostatin^+^ δ cell	
Panel 3 in Fig. 3	Insulin^+^ β cell	VCAM-1	G3BP1	Glucagon^+^ α cell	ICAM-1	Cleaved caspase 3
Panel 4 in Fig. 4	ICA512		Trypsin	Insulin receptor β	Insulin receptor α	CK19
Panel 5 in Figs. 5 and 6	Insulin^+^ β cell	α-SMA	SARS-CoV-2 S1 protein	Ki67	COL1A1	CD31
Panel 6 in Supplemental Fig. 1	Insulin^+^ β cell	Glucagon^+^ α cell		NRP1	ACE2	TMPRSS2

### Quantitative analysis of multiplex immunofluorescence staining

Firstly, each slide was scanned by the Akoya Vectra Polaris state-of-the-art multispectral imaging system, then, we used Phenochart slide viewer software to assist in viewing and navigating whole slide scans, selecting areas for further review, annotating regions for analysis, or selecting stamps for MSI. To view acquired images, click the Login button in the upper right-hand corner. Type in a username and press OK. Next, select Load Slide in the upper left-hand corner. Then, we need to select annotated regions for inForm Projects. We used “Stamps” to select regions. Nine to ten representative regions were selected in each group. Choose “Acquisition” to take multispectral fields of the stamp area the next time this slide is on the Vectra™; choose “Review” to create stamps that must be reviewed before they can be acquired. Vectra will only acquire multispectral fields of accepted stamp annotations; choose “Push” to inForm to launch the selected stamp area into inForm™. This option will only allow stamps at the scan’s native resolution; choose inForm Projects to select stamp areas for analysis in inForm; load the.qptiff into inForm 2.4.3 and later analyze these fields. In inForm, we started a new project and unmixing of fluorophores and autofluorescence, once all images have been prepared, we moved on to the Segment Tissue button by selecting the button at the top workflow schematic. Under Segment Tissue, and in particular, the Trainable Tissue Segmentation module, the program will utilize a user-trainable algorithm for tissue segmentation based on morphology as well as specified markers. Most time, we use DAPI-stained nuclei as the parameter of cell segmentation. We selected adaptive cell segmentation and set phenotyping. After training different regions and setting parameters, I scored each image and exported these data from inForm project. These original data were opened and further calculated in Excel and designed by Graphpad software.

### In situ hybridization

To examine SARS-CoV-2 genomic RNA in formalin-fixed paraffin-embedded tissues, ISH was performed using the RNAscope® 2.5 HD Reagent Kit-RED (cat. no. 322310; Advanced Cell Diagnostics, Newark, CA) and the ISH Probe-V-nCoV2019-S (cat. co. 848561, positive-sense RNA probe) (genomic RNA fragment 21631-23303, RefSeq #NC_045512.2) as previously described.^65^ Briefly, tissue sections were deparaffinized with xylene, underwent a series of ethanol washes and peroxidase blocking, and were then heated in antigen retrieval buffer and digested by proteinase. Sections were exposed to an ISH target probe and incubated at 40 °C in a hybridization oven for 2 h. After rinsing, the ISH signal was amplified using a pre-amplifier and amplifier conjugated to alkaline phosphatase and incubated with DAB for visualization at room temperature. Sections were then stained with hematoxylin, air-dried, mounted, and stored at 4 °C until image analysis.

### Enzyme-linked immunosorbent assay

Anti-monkey enzyme-linked immunosorbent assay kits (Wayen Biotechnologies, Shanghai, China) were used to assay scar cytokine levels according to the manufacturer’s instructions.

### Statistical analysis

All assays were performed on three separate occasions. Data were expressed as means ± SD. We have checked the distribution of all datasets, and all were parametric. The collected data were analyzed with GraphPad Prism 8.0 software (GraphPad Software, San Diego, CA).

### Supplementary information

Supplementary information files

## Data Availability

All raw data are available from the corresponding author upon reasonable request. The datasets presented in this study can be found in online repositories. The names of the repository and accession number can be found below: MetaboLights and the unique identifier MTBLS9671 together with the URL www.ebi.ac.uk/metabolights/MTBLS9671.
