# Supplementary material for: Infection with SARS-CoV-2 can cause pancreatic impairment
Source: Signal Transduct Target Ther. 2024 Apr 12;9:98. doi: 10.1038/s41392-024-01796-2 (PMC11014980; doi:10.1038/s41392-024-01796-2)
Supplement: Supplementary file 1 — Supplementary information files [file 41392_2024_1796_MOESM1_ESM.docx]

Supplementary Materials for

Infection with SARS-CoV-2 can cause pancreatic impairment

Wei Deng1^#^, Linlin Bao^1#^, Zhiqi Song^1#^, Ling Zhang^1#^, Pin Yu^1#^, Yanfeng Xu^1^, Jue Wang^2,3^, Wenjie Zhao^1^, Xiuqin Zhang^2,3^, Yunlin Han^1^, Yanhong Li^1^, Jiangning Liu^1^, Qi Lv^1^, Xujian Liang^1^, Fengdi Li^1^, Feifei Qi^1^, Ran Deng^1^, Siyuan Wang^1^, Yibai Xiong^1^, Ruiping Xiao^2,3,4*^, Hongyang Wang^5,6,7*^, Chuan Qin^1,8*^

Correspondence to: xiaor@pku.edu.cn, hywangk@vip.sina.com, qinchuan@pumc.edu.cn

**This PDF file includes:**

Materials and Methods pg 2-5

Figures. S1 to S5 pg 6-12

**Materials and Methods**

Proteomics Analysis

*Protein Extraction*

Samples were sent to Metware Biotechnology Co.,Ltd (Wuhan, China) for proteomics analysis. After thawed, samples were lyophilized and concentrated to about 2ml by vacuum concentration meter. Add 4times volume of precooled acetone to the samples and still at -20℃ for 2h. After centrifugation, the protein pellets were air-dried and resuspended in 8M urea/100mM TEAB (pH 8.0). Proteins were reduced with 10mM DTT at 56℃ for 30 min, alkylated with 50mM iodoacetamide (IAM) at room temperature for another 30 min in the dark. Add 4 times volume of precooled acetone and still at -20℃ for 2h. After centrifugation, the protein pellets were air-dried and resuspended in 8M urea/100mM TEAB (pH 8.0). Total protein concentration was measured by BCA protein quantitation assay.

*Trypsin Digestion*

Equal amount of proteins from each sample （~100μg）were used for tryptic digestion. Trypsin was added at an enzyme-protein ratio of 1:50 (w/w), the digest reaction was performed at 37℃ for 12-16 hours. After digestion, peptides were desalted using C18 Cartridge followed by drying with Vacuum concentration meter.

*LC-MS/MS Analysis*

  Liquid chromatography (LC) was performed on a nanoElute UHPLC (Bruker Daltonics, Germany). About 200 ng peptides were separated within 60 min at a flow rate of 0.3 uL/min on a commercially available reverse-phase C18 column with an integrated CaptiveSpray Emitter (25cm x 75μm ID, 1.6μm, Aurora Series with CSI, IonOpticks, Australia). The separation temperature was kept by an integrated Toaster column oven at 50℃. Mobile phases A and B were produced with 0.1 vol.-% formic acid in water and 0.1% formic acid in ACN. Mobile phase B was increased from 2 to 22% over the first 45 min, increased to 35% over the next 5 min, further increased to 80% over the next 5 min, and then held at 80% for 5 min.

  The LC was coupled online to a hybrid timsTOF Pro2 (Bruker Daltonics, Germany) via a CaptiveSpray nano-electrospray ion source (CSI). To establish the applicable acquisition windws for diaPASEF mode, the timsTOF Pro2 was operated in Data-Dependent Parallel Accumulation-Serial Fragmentation (PASEF) mode with 10 PASEF MS/MS frames in 1 complete frame. The capillary voltage was set to 1400 V, and the MS and MS/MS spectra were acquired from 100 to 1700 m/z. As for ion mobility range (1/K0), 0.7 to 1.4 Vs/cm^2^ was used. The TIMS accumulation and ramp time were both set to 100ms, which enable an operation at duty cycles close to 100%. The “target value” of 10,000 was applied to a repeated schedule, and the intensity threshold was set at 2500. The collision energy was ramped linearly as a function of mobility from 59 eV at 1/K0 = 1.6 Vs/cm^2^ to 20 eV at 1/K0 = 0.6 Vs/cm^2^. The quadrupole isolation width was set to 2Th for m/z < 700 and 3Th for m/z > 800.

  In diaPASEF mode, the instrument control software was extended to define quadrupole isolation windows as a function of the TIMS scan time. Seamless and synchronous ramping of all applied voltage is achieved by modifying the instrument control electronics. We defined 25 Th isolation windows from m/z about 400 to 1200 and totally 64 windows were defined. Other parameters were the same as DDA-PASEF mode.

*Database search and quantification*

  MS raw data were analyzed using DIA-NN(v1.8.1) with library-free method. the Homo sapiens SwissProt database(20425 entries) was uesed to creat a spectra library with deep learning algrithms of neural networks. the option of MBR was employed to create a spectral library from DIA data and then reanlyse using this library. FDR of search results was adjusted to < 1% at both protein and precursor ion levels, the remaining identifications were used for further quantification analysis.

Lipidomics and Metabonomics Analyses

Serum preparation and extraction

*Hydrophilic compound extraction*

Samples were sent to Metware Biotechnology Co.,Ltd (Wuhan, China) for widely targeted lipidomics and metabonomics analyses. Samples were thawed on ice, vortexed for 10 s, and mixed well. Then, 300 µL of pure methanol was added to 50 µL of serum, the mixture was vortexed for 3 min, and centrifuged at 12,000 rpm at 4℃ for 10 min. The supernatant was collected and centrifuged at 12,000 rpm at 4℃ for 5 min. The sample was left in a refrigerator at -20℃ for 30 min, centrifuged at 12,000 rpm at 4℃ for 3 min, and 150 µL of supernatant was placed in the liner of the corresponding injection bottle for on-board analysis.

*Hydrophobic compound extraction*

Samples were thawed on ice, vortexed for approximately 10 s, and then centrifuged at 3000 rpm at 4℃ for 5 min. Each sample (50 µL) was homogenized with 1 mL of the mixture (including methanol, MTBE, and internal standard mixture), and vortexed for 15 min. Then, 200 µL of water was added, the mixture was stirred for 1 min and centrifuged at 12,000 rpm at 4℃ for 10 min, and 500 µL of the supernatant was kept and lyophilized. The powder was dissolved with 200 µL of mobile phase B, and then stored at −80℃. Finally, the dissolved solution was placed in a sample bottle for LC-MS/MS analysis.

UPLC conditions of hydrophilic compounds

*T3 UPLC Conditions*

Sample extracts were analyzed using LC-ESI-MS/MS (ExionLC AD, Sciex, Framingham, MA; QTRAP® System, Sciex). The UPLC conditions were as follows: column, ACQUITY UPLC HSS T3 C18 (1.8 µm, 2.1 mm × 100 mm; Waters, Mississauga, Canada); column temperature, 40℃; flow rate, 0.4 mL/min; injection volume, 2 μL; solvent system, water (0.1% formic acid):acetonitrile (0.1% formic acid); gradient program, 95:5 V/V at 0 min, 10:90 V/V at 11.0 min, 10:90 V/V at 12.0 min, 95:5 V/V at 12.1 min, 95:5 V/V at 14.0 min.

*Amide UPLC Conditions*

Sample extracts were analyzed using the same LC-ESI-MS/MS system as described earlier. The UPLC conditions were as follows: column, ACQUITY UPLC BEH Amide (1.7 µm, 2.1 mm × 100 mm; Waters); column temperature, 40℃; flow rate, 0.4 mL/min; injection volume, 2 μL; solvent system, water (20 mM ammonium formate and 0.4% ammonia):acetonitrile; gradient program, 10:90 V/V at 0 min, 40:60 V/V at 9.0 min, 60:40 V/V at 10.0 min, 60:40 V/V at 11.0 min, 10:90 V/V at 11.1 min, 10:90 V/V at 15.0 min.

UPLC conditions of hydrophobic compounds

The sample extracts were analyzed using the same LC-ESI-MS/MS system as described earlier. The UPLC conditions were as follows: column, Thermo Accucore™ C30 (2.6 μm, 2.1 mm × 100 mm; Thermo Scientific, Waltham, MA); solvent system, A: acetonitrile/water (60/40,V/V, 0.1% formic acid, 10 mmol/L ammonium formate), B: acetonitrile/isopropanol (10/90 V/V, 0.1% formic acid,10 mmol/L ammonium formate); gradient program, A/B (80:20, V/V) at 0 min, 70:30 V/V at 2.0 min, 40:60 V/V at 4 min, 15:85 V/V at 9 min, 10:90 V/V at 14 min, 5:95 V/V at 15.5 min, 5:95 V/V at 17.3 min, 80:20 V/V at 17.3 min, 80:20 V/V at 20 min; flow rate, 0.35 mL/min; temperature, 45℃; injection volume, 2 μL. The effluent was alternatively connected to an ESI triple-quadrupole (QQQ) linear ion trap (LIT) mass spectrometer (QTRAP).

ESI-QTRAP-MS/MS of hydrophilic compounds

T3 and amide have the same MS parameters.

LIT and QQQ scans were acquired on a QTRAP system (Sciex) equipped with an ESI Turbo Ion-Spray interface, operating in positive and negative ion modes and controlled by Analyst 1.6.3 software (Sciex). The ESI source operation parameters were as follows: source temperature, 500℃; ion spray voltage (IS), 5500 V (positive) and -4500 V (negative); ion source gas I (GSI), gas II (GSII), and curtain gas (CUR) were set at 55, 60, and 25.0 psi, respectively; and the collision gas (CAD) was high. Instrument tuning and mass calibration were performed with 10 and 100 μmol/L polypropylene glycol solutions in the QQQ and LIT modes, respectively. A specific set of MRM transitions was monitored for each period, according to the metabolites eluted within this period.

ESI-QTRAP-MS/MS of hydrophobic compounds

LIT and QQQ scans were acquired on a QTRAP system (Sciex) equipped with an ESI Turbo Ion-Spray interface, operating in positive and negative ion modes and controlled by Analyst 1.6.3 software (Sciex). The ESI source operation parameters were as follows: ion source, turbo spray; source temperature, 500℃; IS, 5500 V (positive) and -4500 V(negative); GS1, GS2, and CUR were set at 45, 55, and 35 psi, respectively; and the CAD was medium. Instrument tuning and mass calibration were performed with 10 and 100 μmol/L polypropylene glycol solutions in the QQQ and LIT modes, respectively. QQQ scans were acquired as MRM experiments with the CAD (nitrogen) set at 5 psi. De-clustering potential (DP) and collision energy (CE) for individual MRM transitions were performed with further optimization of DP and CE. A specific set of MRM transitions was monitored for each period, according to the metabolites eluted within this period.

Metabolome analysis

*Principal component analysis*

Unsupervised principal component analysis (PCA) was performed using the statistical function prcomp within R (www.r-project.org). The data were unit variance-scaled before the unsupervised PCA.

*Hierarchical cluster analysis and Pearson’s correlation coefficients*

Hierarchical cluster analysis (HCA) results of samples and metabolites were presented as heatmaps with dendrograms, while Pearson’s correlation coefficients (PCC) between samples were calculated using the cor function in R and presented as only heatmaps. Both HCA and PCC were carried out using the R package ComplexHeatmap. For HCA, normalized signal intensities of metabolites (unit variance scaling) were visualized as a color spectrum.

*Selection of differential metabolites*

Significantly regulated metabolites between groups were determined by VIP ≥ 1 and absolute log2FC ≥ 1. VIP values were extracted from the results of orthogonal projections to latent structures discriminant analysis (OPLS-DA), which also contain score plots and per-mutation plots, and were generated using the R package MetaboAnalystR. The data were log2-transformed and mean-centered before OPLS-DA. To avoid overfitting, a permutation test (200 permutations) was performed.

*KEGG annotation and enrichment analysis*

Identified metabolites were annotated using the KEGG Compound database (http://www.kegg.jp/kegg/compound/) and then mapped to the KEGG Pathway database (http://www.kegg.jp/kegg/ pathway.html) (Kanehisa, M.; "Post-genome Informatics", Oxford University Press (2000)). Significantly enriched pathways were identified with a hypergeometric test p-value for a given list of metabolites.

**
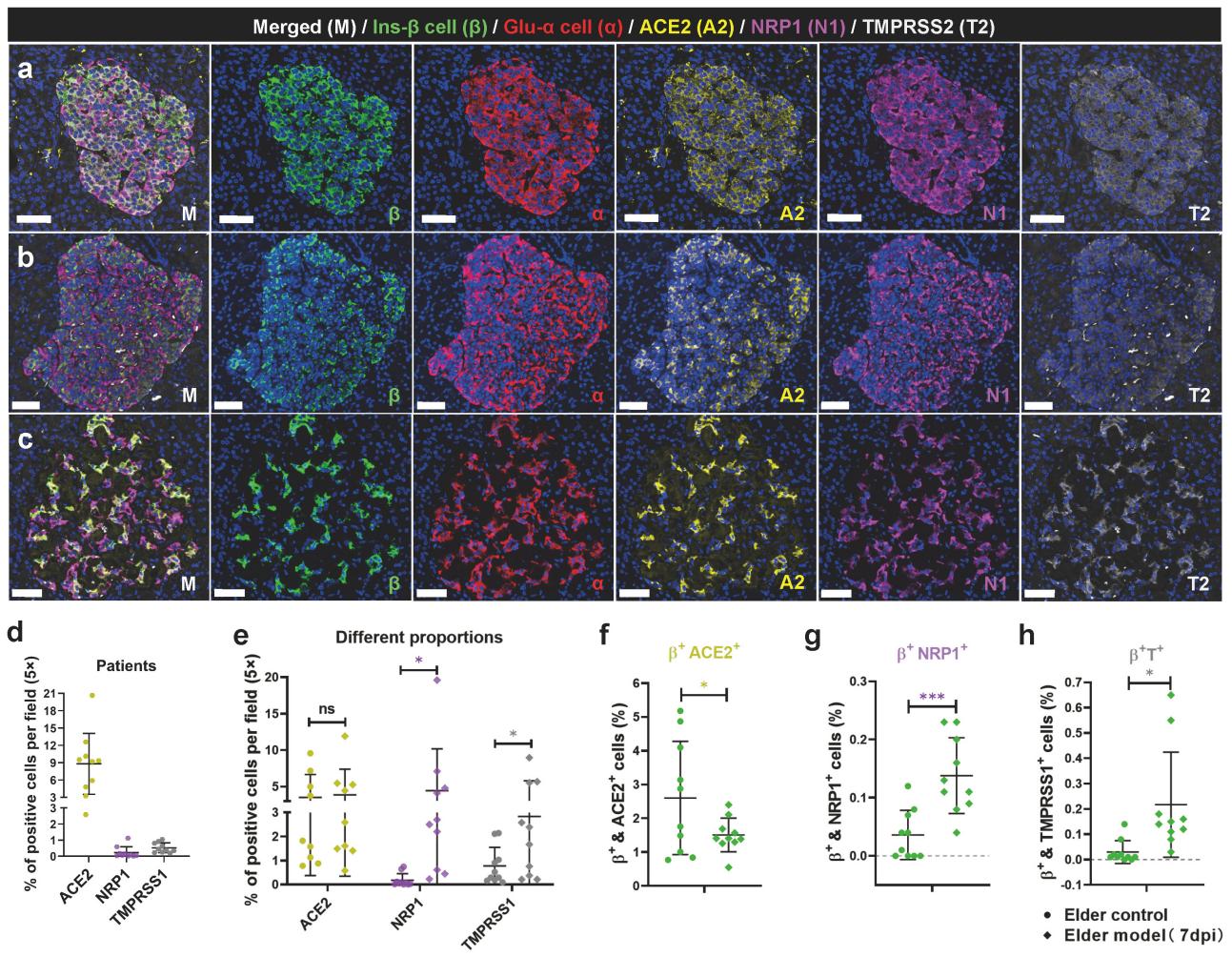
**

**Supplemental Figure 1. Distribution of SARS-CoV-2 mainly receptors in the pancreas of patients and nonhuman primates (NHPs).**

1. Representative multi-labels IF image in the pancreas from the COVID-19 patient autopsy samples were stained for insulin (β, green), glucagon (α, red), ACE2 (A2, yellow), NRP1 (N1, magenta), and TMPRSS2 (T2, white). Scale bars, 50μm.
2. Representative multi-labels IF image in the pancreas from the elder control NHP samples were stained for the same multi-labels panel. Scale bars, 20μm.
3. Representative multi-labels IF image in the pancreas from the elder COVID-19 NHP model samples were stained for the same multi-labels panel. Scale bars, 20μm.

(d and e) Quantification of the percentage of ACE2^+^ cell, NRP1^+^ cell, and TMPRSS1^+^ cell in the pancreas from the COVID-19 patients and elder model NHPs. (n=10 images examined in total / group).

(f-h) Quantification of the percentage of insulin^+^ACE2^+^ cell, insulin^+^NRP1^+^ cell and insulin^+^ TMPRSS1^+^ cell in the elder control NHPs and elder COVID-19 model NHPs, (n=10 images examined in total / group). Data are presented as mean ± SD. p values were calculated by unpaired two-tailed Student’s t test. **p < 0.05, **p < 0.01,* and ****p < 0.001.*

**
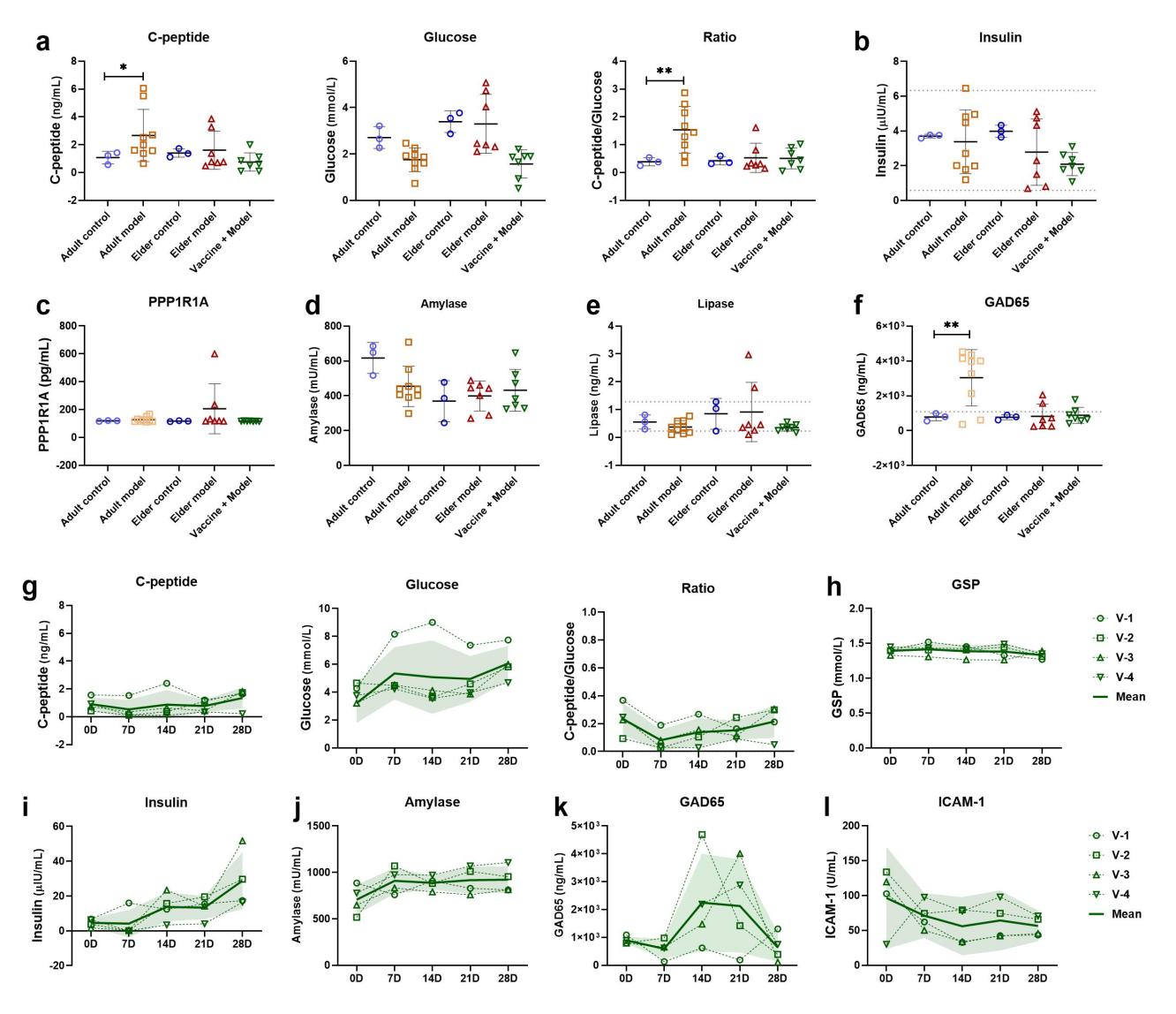
**

**Supplemental Figure 2. Characteristics of Clinical pathological data from adult and elder COVID-19 models and vaccinated COVID-19 NHPs.**

1. C-peptide concentration, glucose concentration, C-peptide/glucose ratio in the serum after overnight fasting from adult control (n=3), adult COVID-19 model (n=9), elder control (n=3), elder COVID-19 model (n=7), and vaccinated COVID-19 NHPs (n=7). One data point per NHP, representing the average of two technical replicates.

(b-f) Serum from different groups the same as in (A) were examined and analyzed by ELISA, targeting indicators known to modulate blood glucose homeostasis or related to pancreas impairment, including insulin, PPP1R1A, amylase, lipase, and GAD65.One data point per NHP, representing the average of two technical replicates. Data are presented as mean ± SD. p values were calculated by unpaired two-tailed Student’s t test. **p < 0.05* and ***p < 0.01.*

(g-l) The Changing trend of the concentrations of C-peptide and glucose, the ratio of C-peptide / glucose, the concentration of GSP, insulin, amylase, GAD65, and ICAM-1 in serum before vaccination and from one week to four weeks post vaccination of four NHPs.

*

*

**Supplemental Figure 3. The similarities and the differences of proteomics and overall metabolomics among adult and elder COVID-19 models and vaccinated COVID-19 NHPs.**

1. The summary of total significant different proteins, metabolites, and lipids were compared in different groups. Red or orange represents up-regulated proteins or metabolites, blue or green represents down-regulated proteins or metabolites.
2. Venn diagram of proteomics, metabolomics and lipidomics among control vs. adult NHPs infected with the prototypic SARS-CoV-2 strain (Adult-prototype), control vs. adult NHPs infected with delta strain (Adult-Delta), control vs. elder NHPs infected with the prototypic SARS-CoV-2 strain for 3 days (Elder-prototype-3DPI) and control vs. elder NHPs infected with the prototypic SARS-CoV-2 strain for 7 days (Elder-prototype-7DPI).

Venn diagram of proteomics, metabolomics and lipidomics among control vs. Adult-prototype, control vs. Adult-Delta, control vs. Elder-prototype-3DPI, control vs. Elder-prototype-7DPI, control vs. adult NHPs had vaccinated and infected with delta strain (Vaccine-Delta), control vs. adult NHPs had vaccinated and infected with the prototypic SARS-CoV-2 strain (Vaccine-prototype). Pro, the prototypic SARS-CoV-2 strain.

**
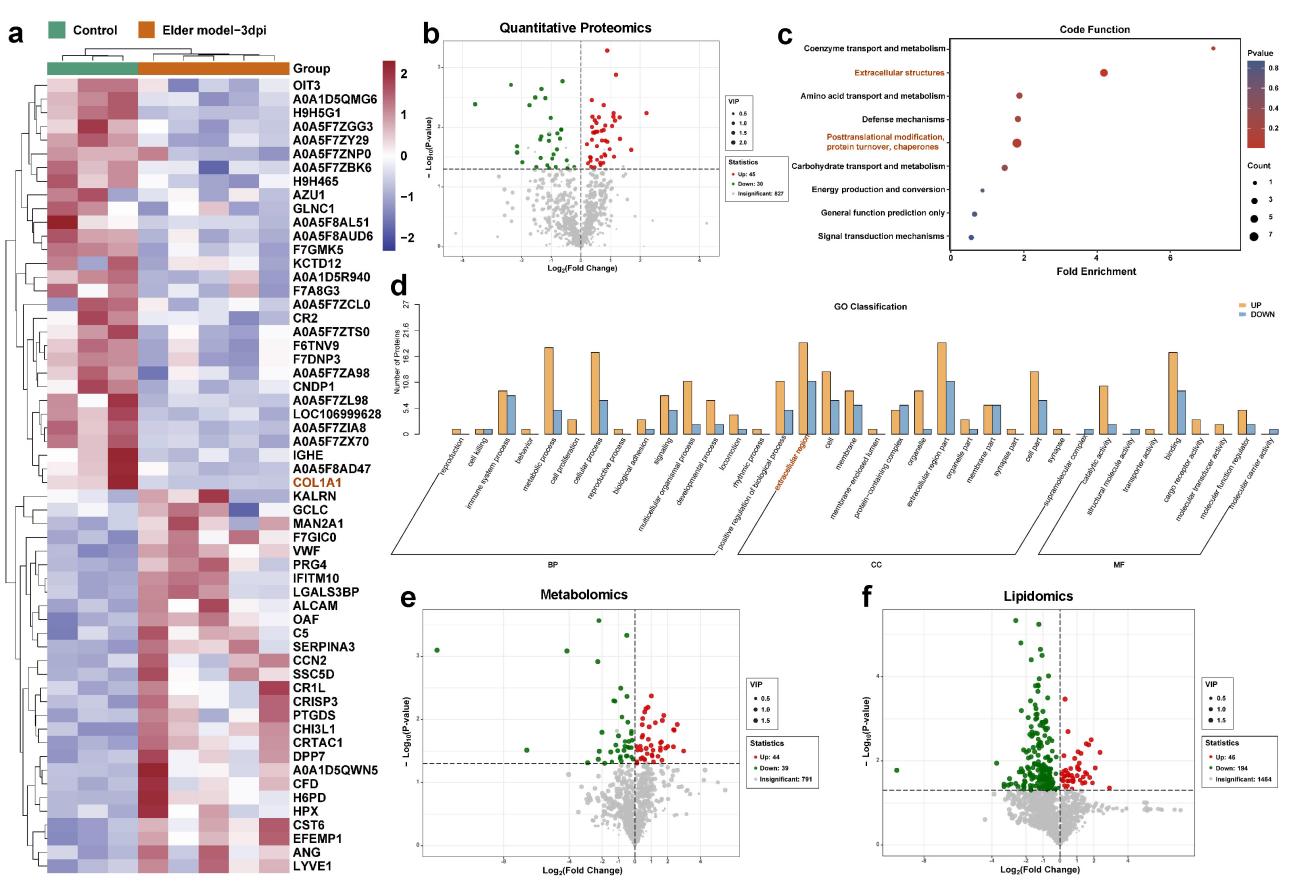
**

**Supplemental Figure 4. The characteristic of proteomics, metabolomics, and lipidmics of elder COVID-19 NHPs model at 3 day post infection (Elder model-3dpi) compared these of control group.**

1. Heatmap reveals the classification of control and elder model-3dpi (x-axis) according to differentially expressed protein (y-axis) and unbiased clustering analysis. Every row represents a protein, and every column represents a serum sample.
2. Volcano plot showing the relative content differences of proteins between control group and elder model-3dpi group and the significance of statistical differences. Each dot in the volcano plot represents a protein, with green dots representing downregulated differential proteins, red dots representing upregulated differential proteins, and gray dots representing proteins detected but not significantly different.
3. Bubble diagram of the cluster of orthologous groups of proteins (KOG) analysis between control group and elder model-3dpi group. From the KOG enrichment analysis results of different proteins, 9 functional classifications with top Pvalue ranking (ranked from small to large) were selected to draw the bubble diagram of enrichment items.
4. Bar chart of gene ontology (GO) classification. GO is divided into three parts: biological process (BP), cellular component (CC), and molecular function (MF).

(e and f) Volcano plot showing the relative content differences of metabolites (e) and lipids (f) between control group and elder model-3dpi group and the significance of statistical differences. Each dot in the volcano plot represents a metabolites or lipids, with green dots representing downregulated differential metabolites or lipids, red dots representing upregulated differential metabolites or lipids, and gray dots representing metabolites or lipids detected but not significantly different.

**
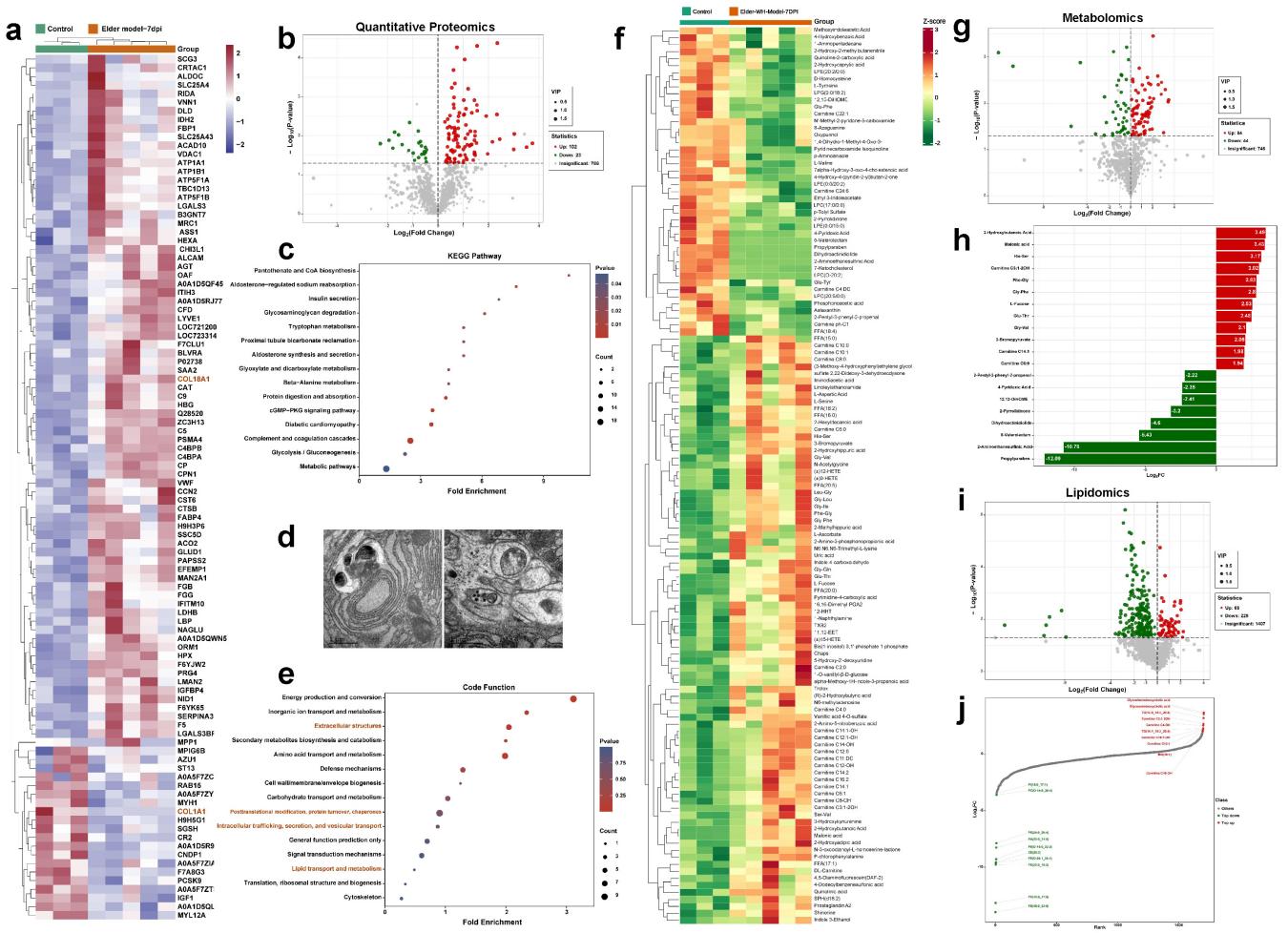
**

**Supplemental Figure 5. The characteristic of proteomics, metabolomics, and lipidmics of elder COVID-19 NHPs model at 7 day post infection compared these of control group.**

1. Heatmap reveals the classification of control and elder model-7dpi (x-axis) according to differentially expressed protein (y-axis) and unbiased clustering analysis. Every row represents a protein, and every column represents a serum sample.
2. Volcano plot showing the relative content differences of proteins between control group and elder model-7dpi group and the significance of statistical differences. Each dot in the volcano plot represents a protein, with green dots representing downregulated differential proteins, red dots representing upregulated differential proteins, and gray dots representing proteins detected but not significantly different.
3. Bubble diagram of the Proteome KEGG enrichment analysis of serum. KEGG pathway analysis of differentiating proteins in elder model-7dpi.
4. Transmission electron microscopy observation revealed that the endoplasmic reticulum and mitochondria were damaged.
5. Bubble diagram of the cluster of orthologous groups of proteins (KOG) analysis between control group and elder model-7dpi group. From the KOG enrichment analysis results of different proteins, 15 functional classifications with top Pvalue ranking (ranked from small to large) were selected to draw the bubble diagram of enrichment items.
6. Heatmap reveals the classification of control and elder model-7dpi (x-axis) according to differentially expressed metabolites (y-axis) and unbiased clustering analysis. Every row represents a metabolite, and every column represents a serum sample.
7. Volcano plot showing the relative content differences of metabolites between control group and elder model-7dpi group and the significance of statistical differences. Each dot in the volcano plot represents a metabolite, with green dots representing downregulated differential metabolites, red dots representing upregulated differential metabolites, and gray dots representing metabolites detected but not significantly different.
8. Bar chart revealed the top 20 differential metabolites. Each bar represents a substance, the green bar represents the substance that is down, and the red dot represents the substance that is up.
9. Volcano plot showing the relative content differences of lipids between control group and elder model-7dpi group and the significance of statistical differences. Each dot in the volcano plot represents a lipid, with green dots representing downregulated differential lipids, red dots representing upregulated differential lipids, and gray dots representing lipids detected but not significantly different.
10. Dynamic distribution of lipids. The abscissa represents the cumulative number of substances in order of the difference multiple from small to large, and the ordinate represents the pair value with the difference multiple as base 2. Each dot represents a substance, the green dot represents the substance that is in the top 10 ranking down, and the red dot represents the substance that is in the top 10 ranking up.
